# $\beta$-plane correction for eddy detection and the drivers of eddy activity heterogeneity in a semi-closed maritime continent basin

**DOI:** 10.1038/s41598-026-43244-x

**Published:** 2026-03-31

**Authors:** Gandhi Napitupulu, Kadek Krisna Yulianti, Aditya Rakhmat Kartadikaria, Ivonne Milichristi Radjawane, M. Apdillah Akbar, Maya Eria Sinurat, Amir Yarkhasy Yuliardi, Ejria Saleh, Angelo Constancia Macario, Faruq Khadami, Muhammad Ridwan Ramadhan, Moses Napitupulu

**Affiliations:** 1 Coastal Hazards and Energy System Science Laboratory, Graduate School of Innovation and Practice for Smart Society, Hiroshima University, Hiroshima, Japan; 2 Environmental and Applied Oceanography Research Group, Faculty of Earth Sciences and Technology, Bandung Institute of Technology, Bandung, West Java Indonesia; 3 Study Program of Oceanography, Faculty of Earth Sciences and Technology, Bandung Institute of Technology, Cirebon, West Java Indonesia; 4 Indonesian Agency for Meteorology, Climatology and Geophysics (BMKG), Jakarta, Indonesia; 5 Master Study Program of Earth Science, Faculty of Earth Sciences and Technology, Bandung Institute of Technology, Bandung, West Java Indonesia; 6 Center for Coastal and Marine Area Development, Bandung, West Java Indonesia; 7 Korea-Indonesia Marine Technology Cooperation Research Center, Cirebon, West Java, Indonesia; 8 Department of Marine Science, Faculty of Fisheries and Marine Science, Jenderal Soedirman University, Purwokerto, Central Java Indonesia; 9 Borneo Marine Institute, Universiti Malaysia Sabah, Kota Kinabalu, Sabah Malaysia; 10 Graduate School of Integrated Sciences for Life, Hiroshima University, Higashi-Hiroshima City, Japan; 11 College of Fisheries and Allied Sciences, Zamboanga State College of Marine Sciences and Technology, Zamboanga City, Philippines; 12 Study Program of Naval Architecture and Marine Engineering, Faculty of Engineering, University of Indonesia, Depok, Indonesia

**Keywords:** $\beta$-plane, Eddy current circulation, Indonesian seas, Mesoscale eddies, Okubo–Weiss, Winding angle, Climate sciences, Ocean sciences

## Abstract

The Indonesian seas, located within the Maritime Continent, are characterized by complex topography, narrow straits, and some of the warmest sea surface temperatures (SSTs) globally. Eddy activity in this region is strongly influenced by monsoonal winds, the Indonesian Throughflow (ITF), Intertropical Convergence Zone (ITCZ), and local bathymetry, producing seasonal patterns that differ from those of the open Pacific and Indian Oceans. Most existing eddy detection methods, however, were designed for mid-latitude regions and neglect equatorial corrections to the Coriolis parameter. This study introduces a localized $\beta$-plane correction to improve Coriolis parameter estimation for eddy detection in equatorial waters, applied to three decades (1993–2022) of satellite-derived sea level anomaly data. Using a modified Winding Angle (WA) method, we identified 8,435 anticyclonic eddies (AEs) and 8,126 cyclonic eddies (CEs), with 7,656 AEs and 7,415 CEs confirmed as persistent features based on the Okubo*-*Weiss (OW) parameter. Eddy sizes were predominantly 60–80 km, with mesoscale eddies concentrated in the northern sector of the domain and along its eastern and southern boundaries. Seasonal variability was evident: AEs peaked during the southeast monsoon (June–August), while CEs dominated during the northwest monsoon (December-February) with stable size. During the eddy-minimum season, high-vorticity CEs formed at lower latitudes than AEs. Regionally, the Java Sea and North Natuna Sea hosted the most energetic eddies during the southeast monsoon, whereas the Sulu, Celebes, Maluku, and Banda Seas emerged as key eddy formation zones. Although eddies were generally short-lived (21–28 days), they contributed substantially to regional energy variability. The spatio–temporal heterogeneity of eddy activity reflects the combined influence of monsoonal wind forcing, SST gradients, and bathymetric constraints, which jointly regulate eddy generation, propagation, and decay. Eddy occurrence also exhibits a hemispheric asymmetry that coincides with ITCZ displacement. These results underscore the coupled atmosphere–ocean processes shaping mesoscale variability in one of the world’s most dynamic tropical marine environments.

**Supplementary Information:**

The online version contains supplementary material available at 10.1038/s41598-026-43244-x.

## Introduction

The Maritime Continent (MC), a region of Southeast Asia situated between the Indian and Pacific Oceans, encompasses Indonesia, Malaysia, the Philippines, Papua New Guinea, Timor-Leste, and parts of southern Thailand and Vietnam. Its intricate geography of islands, peninsulas, and semi-enclosed seas exerts a strong influence on global climate, ocean circulation, and monsoon systems^1^. Mesoscale eddies frequently form throughout this region and contribute to heat redistribution, water mass exchange, and atmosphere–ocean coupling^2^. Understanding these eddies is central to clarifying the mechanisms that shape spatial heterogeneity in circulation within semi-enclosed basins such as the Indonesian Seas.

In low-latitude environments like the Indonesian Seas, the Coriolis parameter ($f$) is small yet varies rapidly with latitude, which heightens the sensitivity of mesoscale processes to minor meridional shifts^3^. This characteristic presents limitations for the traditional $f$-plane approximation that assumes a constant $f$ and is more appropriate at mid and high latitudes. In contrast, equatorial basins experience sharp changes in $f$ over distances of only tens to hundreds of kilometers, making the $\beta$-plane approximation a more physically meaningful representation of planetary vorticity gradients^4^. Integrating the $\beta$-plane into geostrophic velocity calculations enables the Coriolis term to vary with latitude, improving reconstructed flow fields and the depiction of eddy rotational strength and structure^5^. This refinement is crucial near the equator, where small errors in $f$ can produce large biases in inferred vorticity, intensity, and propagation. The $\beta$-plane correction, therefore, enhances the realism of eddy detection from sea level anomaly (SLA) fields, capturing the influence of meridional gradients of planetary vorticity on mesoscale dynamics in narrow straits and semi-enclosed basins across the MC^6^.

The Indonesian Seas host a rich tropical marine environment with high biodiversity and natural resources^7^. Mesoscale eddies, which manifest as rotating water masses propagating relative to the mean flow^8^, redistribute heat, momentum, and nutrients, thereby shaping marine productivity and upwelling^9^,Lovecchio et al., 2022). These features influence both upper-ocean physical properties and subsurface ecosystem processes^10,11^. Eddy generation arises through interactions between large-scale currents and variable seabed topography as well as through internal instabilities^12,13^. Submarine ridges, seamounts, and deep basins can deflect flows, while wind variability, sea surface temperature (SST) changes, and tidal forcing further modulate eddy activity^14,15^. The combined influence of barotropic and baroclinic instabilities adds additional complexity, especially within narrow passages and shelf-break regions typical of the Indonesian archipelago^16–18^.

Eddy-driven transport strongly shapes SST distribution, biological productivity, vertical mixing, sediment redistribution, and coastal morphology^19^,Lovecchio et al., 2022;^20^. These processes are particularly important in semi-enclosed basins and coastal ecosystems of Indonesia, which are highly sensitive to natural variability and anthropogenic stressors. Improved understanding of eddy pathways and life cycles can support fisheries management, enhance prediction of upwelling events, and strengthen projections of regional circulation under climate variability^21,22^.

Globally, eddy hotspots have been identified in regions of strong boundary currents and upwelling systems, such as the East Australian Current and the Peru and California coasts, as well as in marginal seas like the South and East China Seas (e.g.,^23^). Despite their importance, low-latitude eddies remain comparatively understudied due to earlier observational limitations. Past altimetry missions were better suited to detecting long-lived mid-latitude eddies, whereas short-lived equatorial eddies were often unresolved. Recent advances in high-frequency gridded sea surface height products now allow improved monitoring of transient eddies in tropical basins, enabling more comprehensive investigations in the Indonesian Seas.

The Indonesian archipelago forms the only tropical maritime pathway connecting the Pacific and Indian Oceans through the Indonesian Throughflow (ITF) ^24^,^25^). Along the western ITF pathway, the Mindanao Current splits south of Mindanao Island into branches feeding the North Equatorial Countercurrent (NECC), retroflecting into the Pacific, or entering the Celebes Sea^26,27^. These Mindanao Current source waters contribute not only to the western pathway but also indirectly to the eastern pathway through subsequent redistribution within the interior seas*.* Part of the MC retroflects along northern Sulawesi, while the remaining flow continues southward as the Makassar Strait Throughflow (MST) into the Flores and Banda Seas^28^. In parallel, the eastern ITF pathway is supplied by both redistributed Pacific-origin waters and northern hemisphere (NH) waters entering through the Maluku and Halmahera Seas, which subsequently connect to the Seram and Banda Seas^29^. Additional contributions from the NH also enter the Indonesian Seas via the South China Sea (SCS) through the Mindoro and Karimata Straits^30^. ITF waters exit through the Lesser Sunda Islands and the Timor Passage^31^, producing a complex pattern of circulation regimes that shape the dynamic structure of each sub-basin.

Within this circulation framework, mesoscale eddies interact with the ITF through eddy–mean flow coupling. Although ITF variability across intraseasonal to decadal scales has been widely examined, the role of eddies in modulating intraseasonal transport remains less understood due to fragmented observations and the difficulty of tracking eddies amid strong shear and abrupt bathymetric transitions^32^.

Previous studies reveal strong multiscale temporal variability in Indonesian eddies. For example, 50-day intraseasonal oscillations in horizontal velocity observed at mooring sites in the Sulawesi Sea have been linked to baroclinic Rossby wave resonance^33^. These oscillations, along with their interannual variability, correspond to the release of energetic eddies that renew upper-layer waters in the Sulawesi Sea and Makassar Strait. Numerical experiments indicate recurring eddy activity in the Flores Sea during the austral summer, coincident with reduced ITF transport, while seasonal variability in eddy kinetic energy (EKE) shows depth-dependent periodicities in the Sulawesi Sea and contrasting seasonal behaviors of anticyclonic and cyclonic eddies in the Sulu Sea^34^.

This study advances eddy research in the MC by implementing a localized $\beta$-plane correction in the eddy detection process, a refinement seldom applied in tropical regions. Most previous studies adopt the $f$-plane approximation, but large meridional gradients in $f$ at low latitudes introduce systematic biases (e.g.,^23,35^). Applying the $\beta$-plane in geostrophic velocity computation improves the physical realism of rotational diagnostics, especially in the Makassar Strait, Banda Sea, and Halmahera region, where strong spatial gradients in $f$ amplify sensitivity to its variation. This approach enhances characterization of eddy size, strength, and structure and provides new insight into mesoscale interactions with complex bathymetry and low-latitude circulation pathways. The long-term eddy climatology for 1993–2022 further provides the first basin-wide assessment across the interconnected seas of the MC, forming a comprehensive basis for evaluating links between eddy variability, monsoonal forcing, and ITF dynamics.

Despite these advances, a unified description of the spatial distribution, seasonal variability, and mechanistic drivers of eddies in the Indonesian Seas remains limited. This study addresses this gap by combining long-term satellite altimetry (1993–2022) with tide gauge (TG) validation across key locations of the MC to establish the reliability of satellite-derived sea level variability prior to eddy detection. Building on this validated dataset, we implement a localized $\beta$-plane correction in the computation of geostrophic velocities to improve the dynamical consistency of eddy identification in this low-latitude archipelagic environment. This framework enables a coherent assessment of spatial patterns, seasonal variability, and regional heterogeneity of eddy activity across interconnected sub-basins. Ultimately, this work advances understanding of mesoscale eddy dynamics in the Indonesian Seas and provides a robust observational foundation for improving circulation analyses, ecosystem assessments, and climate variability studies in semi-enclosed tropical basins.

## Materials and methods

### Study area and data

**Fig. 1:**
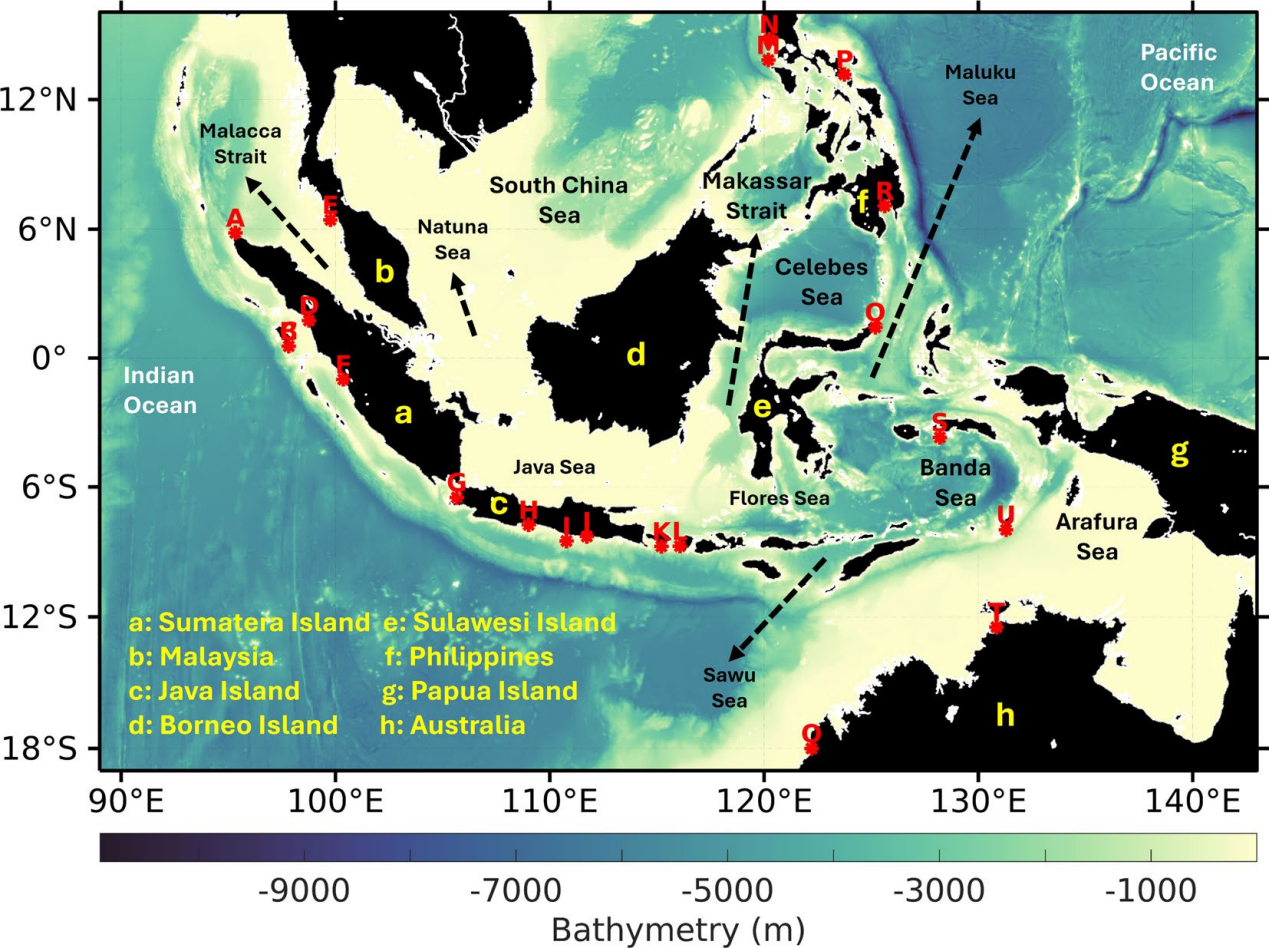
Study area of the Indonesian seas (19°S to 16°N, 89°E to 143°E). Red dots indicate tide gauge (TG) stations used for the validation of satellite-derived sea level anomaly (SLA) data. Bathymetry is from GEBCO 2025. Map created in MATLAB R2023b using M_Map (Pawlowicz, 2020, version 1.4 m, www.eoas.ubc.ca/~rich/map.html).

This study examines mesoscale eddies in the Indonesian seas, a dynamically complex region that regulates ocean–atmosphere interactions within the MC. The analysis domain extends from 19°S to 16°N and 89°E to 143°E (Fig. 1), encompassing major straits, marginal seas, and semi-enclosed basins influenced by both the Pacific and Indian Oceans. The intricate coastline, narrow passages, strong tidal signals, and steep bathymetric gradients make this region particularly challenging for satellite altimetry, while also providing a natural laboratory for examining eddy dynamics and spatial heterogeneity.

Bathymetric information from GEBCO 2025 is used to provide topographic context for interpreting eddy structure and distribution. Sea surface height variability is derived from a Level 4 gridded SLA product with 0.25° $\times$ 0.25° spatial resolution and daily temporal resolution (Copernicus Marine Service, 10.48670/moi-00237). Although along-track altimetry provides direct measurements of sea level, its irregular spatial sampling over the Indonesian archipelago, caused by sparse ground tracks and the presence of narrow straits, limits its suitability for systematic eddy detection. The multi-mission gridded SLA product, generated through optimal interpolation and cross-calibration of along-track data, offers spatially continuous fields required for automated eddy identification and tracking. However, the reliability of these gridded products in coastal and semi-enclosed tropical seas remains uncertain because of tidal contamination, land proximity effects, and interpolation smoothing.

To evaluate whether the satellite-derived SLA fields are sufficiently accurate for eddy detection in Indonesian seas, we conducted a systematic comparison with in situ sea level observations from TG stations provided by the University of Hawaii Sea Level Center (UHSLC, https://uhslc.soest.hawaii.edu/data/). Daily TG records, indicated by red dots in Fig. 1, were collocated with the nearest satellite grid points to assess statistical agreement. Correlation coefficients ($r$) and root mean square error ($RMSE$) were calculated to quantify performance across different coastal and semi-enclosed environments. This validation step is necessary to assess the ability of the gridded SLA product to represent mesoscale variability accurately. Confirming this capability provides confidence in its use for eddy detection and climatological analyses across the Indonesian Seas.

In addition, rainfall data from ERA5 reanalysis^36^ were used to characterize large-scale atmospheric forcing, particularly the seasonal migration of the ITCZ, providing atmospheric context for interpreting seasonal variations in eddy activity. Together, these datasets establish a validated observational framework for analyzing mesoscale eddies and assessing their spatial and temporal variability throughout the Indonesian archipelago.

### Eddy detection using the winding angle (WA) method

Mesoscale eddies were identified using the Winding Angle (WA) method applied to the 1993 to 2022 SLA dataset. Weekly SLA fields, interpolated from daily observations, were used to reduce high-frequency noise while retaining sufficient temporal resolution to resolve eddy life cycles. This is particularly important in the Indonesian seas, where strong tides and energetic background currents can obscure transient eddy signals at monthly resolution.

The WA method identifies potential eddy centers as local maxima for anticyclonic eddies (AEs) and local minima for cyclonic eddies (CEs). Streamlines derived from geostrophic velocities are examined, and an eddy is confirmed when the absolute WA exceeds 2π. The eddy boundary is defined by the outermost closed streamline, and the eddy radius is computed as $R = \sqrt{A/ \pi }$, where $A$ is the enclosed area.

Compared to threshold-based approaches such as the Okubo*-*Weiss (OW) method, WA more effectively detects coherent rotational structures and is less sensitive to background strain, particularly in regions with strong shear and complex coastlines. WA serves as the primary detection method, while OW is applied as a secondary dynamical filter.

### Refinement of winding angle (WA) eddies using the Okubo-Weiss (OW) parameter

Although the WA method efficiently detects coherent rotating streamlines, it is purely geometric and does not explicitly verify whether the detected structures are dynamically dominated by vorticity. To ensure that WA-identified eddies represent dynamically robust vortices rather than transient or strain-dominated features, the OW parameter was applied as a post-processing filter.

The OW parameter is computed from the geostrophic velocity components $u$ and $v$ as

1 $$W = S_{n}^{2} + S_{s}^{2} - \xi^{2}$$

where $S_{n} = \frac{\partial u}{{\partial x}} - \frac{\partial v}{{\partial y}}$ is the normal strain, $S_{s} = \frac{\partial v}{{\partial x}} + \frac{\partial u}{{\partial y}}$ is the shear strain, and $\xi = \frac{\partial v}{{\partial x}} - \frac{\partial u}{{\partial y}}$ is the relative vorticity.

A grid cell is classified as an eddy core when $W < - W_{0}$, with $W_{0} = 0.2\sigma_{W}$, where $\sigma_{W}$ is the spatial standard deviation of $W$. Under this framework, elliptic regions ($W < - W_{0}$) are vorticity-dominated and represent dynamically stable eddies, hyperbolic regions ($W > - W_{0}$) are deformation-dominated and interpreted as dynamically unstable features, while background regions (${ \mid }W{ \mid } \le W_{0}$) represent transitional flow.

Thus, in this study, unstable eddies are defined as eddy-like structures initially detected by the WA method but located primarily within deformation-dominated regions ($W > - W_{0}$). These structures are removed because they lack sufficient rotational dominance to maintain coherent eddy behavior. By contrast, WA-detected structures that contain a well-defined OW-identified elliptic core are retained as physically meaningful mesoscale eddies.

The purpose of the OW filter is not to improve the detection rate, but to remove marginal, distorted, or short-lived rotating features that lack sustained vorticity dominance. Consequently, the observed reduction in eddy counts after OW filtering reflects the removal of weak or transitional structures rather than a loss of physically meaningful eddies.

Eddy amplitude is determined using Green’s transformation applied to the closed SLA contour:

2 $$G\left( t \right) = \frac{1}{2\pi C}\log {\mid }z_{t} - z{\mid }\frac{\partial z}{{\partial \tau }}d\tau$$

with:

3 $$\frac{\partial z}{{\partial \tau }} = \frac{dx}{{d\tau }} + i\frac{dy}{{d\tau }}$$

The amplitude is then computed as:

4 $$A = - \frac{1}{d}\frac{d}{dt}\left[ {Re\left( {g\left( t \right)} \right)} \right]$$

Eddy trajectories are tracked using the similarity metric (Vortmeyer-Kley et al., 2016):

5 $$X_{{e_{1} ,e_{2} }} = \frac{\Delta X}{{X_{0}^{2} }} + \frac{\Delta R}{{R_{0}^{2} }} + \frac{\Delta \xi }{{\xi_{0}^{2} }}$$

where $X$ represents the spatial separation between eddy centers, $\Delta R$ and $\Delta \xi$ denote changes in eddy radius and vorticity, and $X_{0}$, $R_{0}$, and $\xi_{0}$ are reference scaling parameters.

Mesoscale eddies contribute approximately 80% of the total kinetic energy in the ocean^37^. EKE is computed as:

6 $$EKE = \frac{1}{2}\left( {u^{2} + v^{2} } \right)$$

Additional diagnostic parameters include relative vorticity $\xi$, shear deformation $S_{s}$, stretching deformation $S_{n}$, and divergence $Div = \frac{\partial u}{{\partial x}} + \frac{\partial v}{{\partial y}}$.

### $\beta$*-Plane correction for eddy detection*

To account for the variation of the Coriolis parameter with latitude, a $\beta$-plane approximation was applied to improve geostrophic velocity estimation and streamline reconstruction. On the $\beta$-plane, the Coriolis parameter is expressed as:

7 $$f = f_{0} + \beta \left( {y - y_{0} } \right)$$

where $f_{0} = 2 \Omega sin \phi_{0}$ is the Coriolis parameter at the reference latitude $\phi_{0}$, $\beta = \frac{{2\Omega cos \phi_{0} }}{{R_{E} }}$ is the meridional gradient of $f$, $y$ is the northward distance from the reference latitude $y_{0}$, and $R_{E}$ is Earth’s radius.

The $\beta$-plane correction modifies the geostrophic velocity calculation from SLA as:

8 $$u_{g} = - \frac{g}{f}\frac{\partial \eta }{{\partial y}}, \quad v_{g} = \frac{g}{f}\frac{\partial \eta }{{\partial x}}$$

where $\eta$ is SLA, $g$ is gravitational acceleration, and $f$ is replaced by its $\beta$-plane form. This adjustment is particularly relevant for the study domain, which spans less than 20° in latitude across the MC, where the assumption of a constant $f$ can introduce systematic biases in eddy detection and characterization. Incorporating the $\beta$-plane ensures more accurate velocity fields, streamline geometry, and WA calculations, especially in regions near the equator where $f$ varies rapidly with latitude.

### Kernel density estimation (KDE)

Kernel density estimation (KDE) was used to characterize the spatial distribution, intensity, and seasonal migration of eddies. KDE is a non-parametric approach that constructs a continuous probability density function from discrete observations without assuming a specific functional form. Eddy centers and corresponding intensity or radius values were treated as point events.

For an eddy center at $\left( {x_{i} ,y_{i} } \right)$, the KDE is:

9 $$\hat{f}\left( {x,y} \right) = \frac{1}{{nh_{x} h_{y} }}\mathop \sum \limits_{i = 1}^{n} K\left( {\frac{{x - x_{i} }}{{h_{x} }}} \right)K\left( {\frac{{y - y_{i} }}{{h_{y} }}} \right)$$

where $n$ is the total number of eddies, $K\left( \cdot \right)$ is the kernel function, and $h_{x}$, $h_{y}$ are the bandwidth parameters that control the degree of spatial smoothing in the zonal and meridional directions. A Gaussian kernel was used in this study due to its smoothness and suitability for geophysical data, and the optimal bandwidth was selected based on Silverman’s rule of thumb, modified for spatial data resolution (Davies et al., 2018;^38^).

The KDE maps were computed for the full dataset as well as for seasonal subsets to capture monsoonal variations in eddy generation and migration. This enabled the identification of persistent eddy “hotspots”, regions with consistently high eddy density, and the detection of shifts in eddy activity between northwest and southeast monsoon. Additionally, the KDE results were stratified by eddy polarity (CE and AE) to examine whether each type exhibited distinct spatial and seasonal patterns. The integration of KDE with the WA–OW detection framework enables a direct connection between eddy spatial statistics and the governing dynamical processes. This unified methodology offers a robust framework for quantifying mesoscale variability in the complex, semi-enclosed basins of the MC.

### Intertropical convergence zone (ITCZ)

The ITCZ is characterized by the seasonal migration of rainfall. To detect the location of ITCZ, the rainfall data from ERA5^39^ is used during the period of analysis. The centroid method, following Liao et al.^40^, is applied.

10 $$\theta_{NH} = \frac{{\mathop \smallint \nolimits_{0}^{30} \varphi \times p \times \cos \varphi d\varphi }}{{\mathop \smallint \nolimits_{0}^{30} p \times \cos \varphi d\varphi }}$$

11 $$\theta_{SH} = \frac{{\mathop \smallint \nolimits_{ - 30}^{0} \varphi \times p \times \cos \varphi d\varphi }}{{\mathop \smallint \nolimits_{ - 30}^{0} p \times \cos \varphi d\varphi }}$$

where $\varphi$ is latitude (°), $p$ is precipitation (mm/day) at that latitude, $\phi_{NH}$ represents the precipitation-weighted centroid in the NH, and $\phi_{SH}$ represents the centroid in the southern hemisphere (SH). Since the analysis focuses on the Indonesian region, where the area of the precipitation belt will be wider^41^, the ITCZ extends up to 30°^42,43^. Therefore, the range of ITCZ detection is set from 30°N to 30°S.

## Result

### Comparison of satellite-derived sea level anomalies with tide gauge data

The comparison between satellite altimetry SLA and TG observations shows strong agreement across most stations based on $r$ and $RMSE$ (Supplementary Figure S1). $r$ range from 0.64 to 0.98, with $RMSE$ between 0.05 and 0.13 m. The highest consistency is observed along the southern coast of Java and Bali, particularly at Benoa (0.98, 0.06 m) and Sadeng (0.97, 0.09 m). Other stations with correlations above 0.90 include Cilacap, Tanjung Lesung, Prigi, Lembar, and Darwin (Table 1), indicating high SLA reliability in regions influenced by open-ocean dynamics and strong alongshore currents.

**Table 1:** Comparison of sea level anomaly from satellite with tide gauge in the Maritime Continent (MC).

No	Station	Correlation	RMSE (m)	Lon	Lat	Start	End
A	Sabang	0.74	0.07	95.33	5.83	20/12/2005	31/12/2023
B	Teluk Dalam	0.74	0.08	97.82	0.56	28/08/2008	02/06/2015
C	Sibolga	0.74	0.08	97.82	0.56	27/04/2005	09/08/2020
D	Langkawi	0.80	0.06	98.77	1.75	01/01/1993	16/11/2023
E	Padang	0.88	0.06	99.76	6.43	09/12/2005	01/11/2023
F	Tanjung Lesung	0.92	0.05	100.37	-1.00	01/02/2008	21/09/2012
G	Cilacap	0.91	0.06	105.67	-6.48	02/04/2018	31/12/2023
H	Sadeng	0.97	0.09	109.02	-7.75	20/07/2008	03/04/2016
I	Prigi	0.95	0.07	110.78	-8.50	27/02/2007	12/12/2021
J	Benoa	0.98	0.06	111.73	-8.28	26/01/2006	31/12/2023
K	Lembar	0.95	0.06	115.21	-8.74	24/09/2008	31/12/2023
L	Lubang	0.81	0.07	116.07	-8.73	13/06/2010	31/12/2023
M	Subic Bay	0.83	0.07	120.20	13.82	01/03/2007	31/12/2023
N	Broome	0.82	0.07	120.25	14.77	01/01/1993	31/12/2023
O	Legaspi	0.71	0.09	122.22	-18.00	01/01/1993	31/12/2023
P	Bitung	0.65	0.13	123.75	13.15	04/09/2008	31/12/2023
Q	Davao	0.92	0.06	125.19	1.44	02/01/1998	31/12/2023
R	Ambon	0.64	0.08	125.63	7.08	03/10/2008	01/08/2023
S	Darwin	0.96	0.05	128.20	-3.70	01/01/1993	31/12/2023
T	Saumlaki	0.68	0.10	130.85	-12.47	03/10/2008	02/10/2021

Across all 20 stations, the mean $r$ is 0.84 and the mean RMSE is approximately 0.075 m, confirming robust overall performance of the gridded SLA product across the MC. More than 70% of stations show correlations exceeding 0.80, demonstrating strong consistency between satellite-derived and in situ sea level variability across diverse dynamical regimes.

Lower agreement is found in more complex coastal or island environments. Bitung (0.65, 0.13 m), Saumlaki (0.68, 0.10 m) and Ambon (0.64, 0.08 m) show reduced correspondence, likely due to unresolved local processes such as topographic trapping and river discharge^44^. These reduced correlations do not compromise the eddy detection framework, as eddy identification is performed offshore where satellite performance is stronger.

Spatially, higher correlations occur along open-ocean margins facing the eastern Indian Ocean and Timor Sea, generally exceeding 0.88, whereas semi-enclosed basins such as the Maluku and Banda Seas show more moderate values. This contrast underscores the sensitivity of satellite accuracy to coastal complexity and tidal amplitude. Padang (0.88, 0.06 m) and Langkawi (0.80, 0.06 m) reflect smooth large-scale variability in the eastern Indian Ocean. Philippine stations such as Legaspi (0.71, 0.09 m) show moderate agreement, potentially influenced by monsoonal winds, typhoons, and complex island geometry^45^. Subic Bay and Broome, with correlations of 0.82 to 0.83, further demonstrate that SLA performs well in semi-enclosed tropical settings.

Overall, the evaluation confirms that daily gridded SLA data are suitable for eddy detection. Satellite altimetry provides basin-scale sea level observations with broad spatial coverage and long temporal continuity, whereas TGs represent localized variability^46^. Although along-track altimetry is the only direct sea level measurement, its sparse repeat tracks and complex coastlines limit sampling in the Indonesian Seas. The multi-mission gridded SLA fields, derived through optimal interpolation, provide spatially continuous daily coverage appropriate for mesoscale structure identification across the MC.

With a spatial resolution of 0.25° $\times$ 0.25°, eddy detection is feasible because mesoscale eddies in the Indonesian Seas typically have radii of 25 to 160 km^35^. Although accuracy decreases near coastlines due to land contamination, footprint effects, and bathymetric influences, multi-sensor merging and interpolation reduce these limitations. Consequently, mesoscale features can be reliably identified across most of the region despite greater uncertainty in nearshore zones.

Collectively, these validation results demonstrate that the satellite SLA dataset provides sufficient accuracy, spatial consistency, and temporal coherence to support robust mesoscale eddy detection across Indonesian seas, particularly in offshore and dynamically active regions.

### Eddy detection in the Maritime Continent detected by WA and WA-OW methods

From 1993 to 2022, the WA method detected 40,049 AEs and 43,821 CEs across the MC. To enhance dynamical consistency, these detections were refined using the OW parameter, forming a hybrid WA–OW framework that integrates closed SLA contours with vorticity–strain diagnostics. This refinement distinguishes rotationally dominated vortices from deformation-dominated flow structures.

After applying the OW criterion, the total number of detected features decreased to 32,368 AEs and 30,486 CEs, corresponding to reductions of approximately 19% and 30%, respectively. These reductions do not imply that the excluded features were erroneous; rather, they represent WA-identified structures lacking a well-defined OW vorticity core and are therefore interpreted as weak, transitional, or deformation-dominated features rather than coherent mesoscale eddies.

Eddy trajectories were subsequently reconstructed by linking consecutive detections based on temporal continuity and spatial consistency. This tracking procedure yielded 8,435 AEs and 8,126 CEs. Among these, 7,656 AEs and 7,415 CEs satisfied the OW dynamical stability criterion and persisted for more than two weeks. Only these eddies were retained for subsequent statistical analyses.

**Fig. 2:**
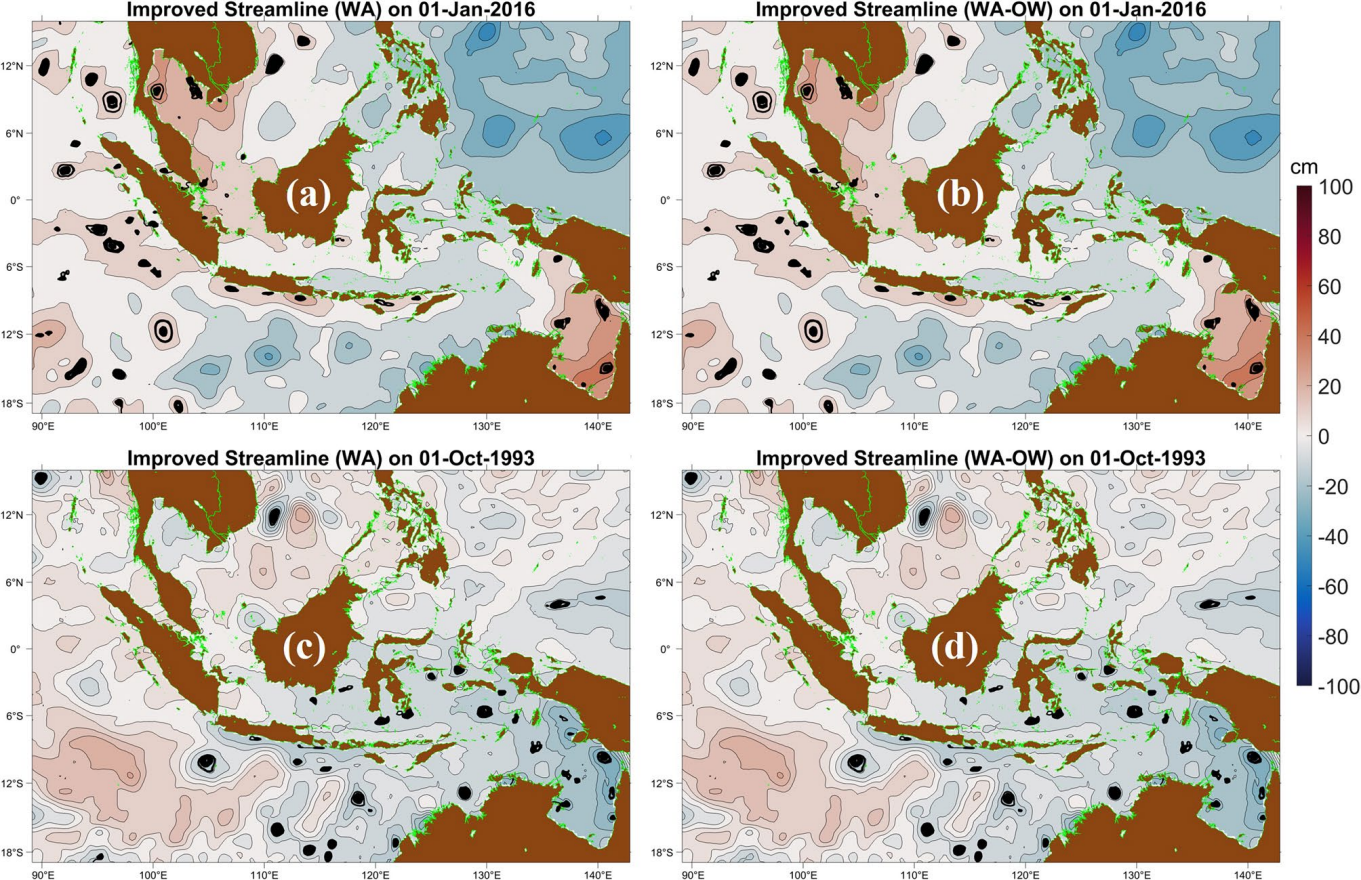
Comparison of eddy detection using the Winding Angle (WA) method and the hybrid WA–Okubo–Weiss (WA–OW) approach on selected dates. (**a**) Anticyclonic eddies (AEs) detected using WA on 1 January 2016; (**b**) the same AE field refined using WA–OW; (**c**) cyclonic eddies (CEs) detected using WA on 1 October 1993; and (**d**) the corresponding CE field refined using WA–OW. The WA–OW method removes deformation-dominated or weakly rotational structures while retaining coherent vortex cores, thereby improving dynamical consistency without eliminating stable eddies. All maps were created in MATLAB R2023b using M_Map (Pawlowicz, 2020, version 1.4 m, www.eoas.ubc.ca/~rich/map.html).

Figure 2 illustrates representative examples of the refinement process. In the western Sumatra region, southern Java, the Malacca Strait, the Natuna Sea, and the Sawu Sea, partially defined or unstable AEs identified by WA are removed after OW filtering (Fig. 2a, b). In contrast, the comparison for CEs (Fig. 2c, d) reveals minimal structural differences, indicating that most WA-detected CEs in this example already exhibit strong rotational dominance. Importantly, the OW filter removes only dynamically inconsistent portions of a structure while preserving coherent vortex cores. This refinement is particularly critical in marginal seas, where complex bathymetry and shear often generate transient features that resemble eddies but lack sustained rotational structure.

**Fig. 3:**
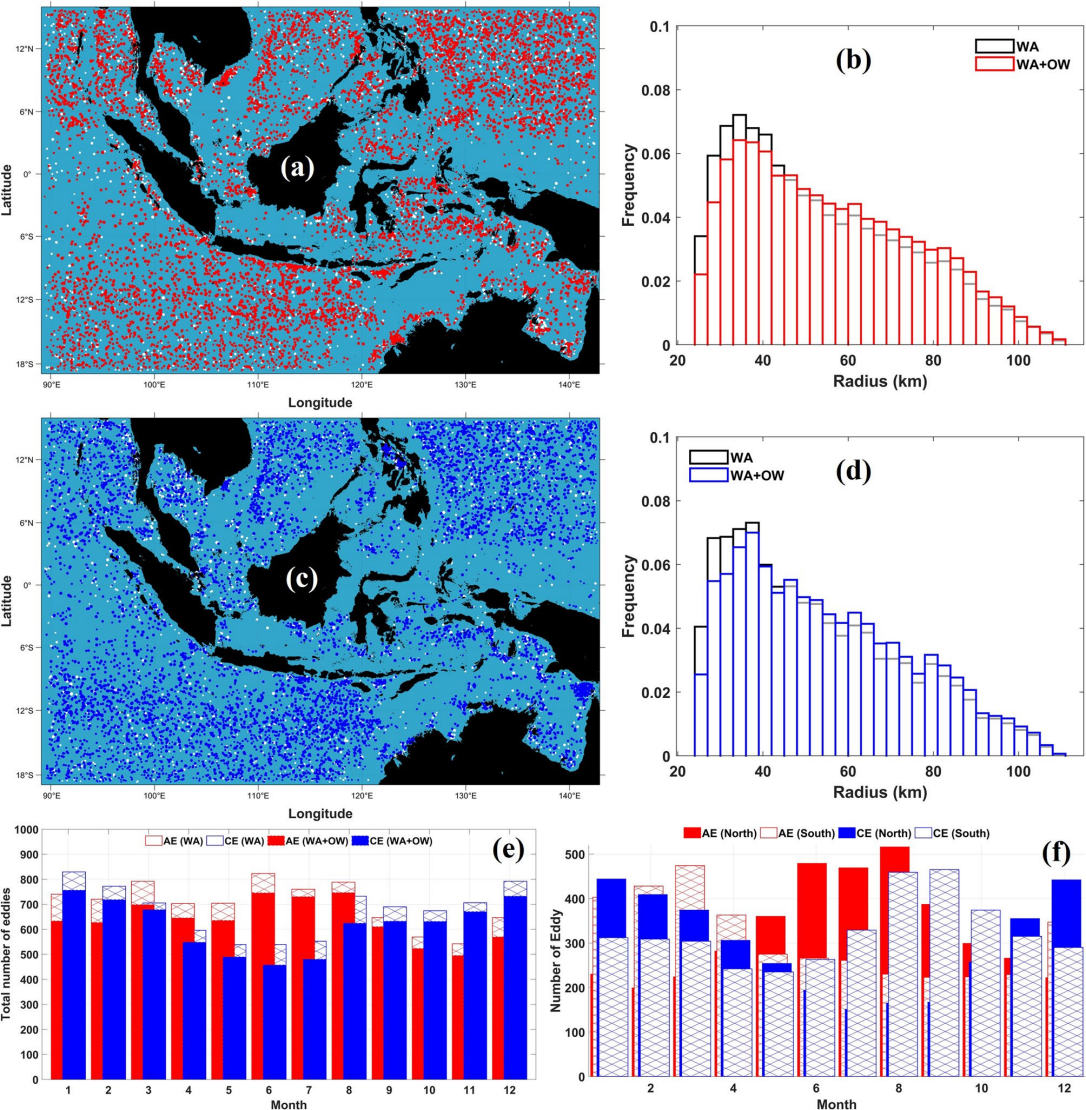
Statistical characteristics of weekly anticyclonic (AE) and cyclonic eddies (CE) detected using the Winding Angle (WA) and WA–Okubo–Weiss (WA–OW) methods from 1 January 1993 to 31 December 2022 across the Maritime Continent (MC). (**a**, **c**) Spatial distribution of AEs and CEs, respectively; red and blue dots indicate retained eddies, while white dots denote features removed by the OW dynamical stability criterion. (**b**, **d**) Probability distributions of eddy radii for AEs and CEs. (**e**) Monthly variation in total AE and CE counts detected by WA and WA–OW methods. (**f**) Monthly variation of AE and CE occurrence in the northern and southern sectors of the MC based on WA–OW detection. Maps in (a, c) were created in MATLAB R2023b using M_Map (Pawlowicz, 2020, version 1.4 m, www.eoas.ubc.ca/~rich/map.html); all panels were generated in MATLAB R2023b.

The spatial distribution and statistical characteristics of AEs and CEs are presented in Fig. 3. Eddy activity is minimal in the Java Sea, consistent with its shallow bathymetry and weak geostrophic flow (Fig. 3a, c). In contrast, high eddy occurrence is observed in the Banda Sea, the SCS, the eastern Indian Ocean, and the western Pacific Ocean. These regions are characterized by strong boundary currents and pronounced Coriolis effects that favor baroclinic instability and mesoscale eddy generation. The spatial heterogeneity of eddy formation therefore reflects the combined influence of regional circulation and bathymetric constraints.

The radii distributions (Fig. 3b, d) show that both AEs and CEs are concentrated within the 30–40 km range. Peak probabilities exceed 0.07 for radii of 33–36 km (AEs) and 36–39 km (CEs). Frequencies decline progressively for larger radii, dropping below 0.01 beyond approximately 90 km and approaching zero above 105 km, indicating the limited occurrence of large mesoscale structures. The WA–OW method preserves the dominant radius band while slightly reducing frequencies at the smallest and largest bins, suggesting that OW filtering removes marginal or weak vortices without altering the intrinsic mesoscale length scale. The consistent unimodal distribution across detection methods indicates that the observed eddy scale is dynamically controlled rather than algorithm dependent.

Monthly variability reveals pronounced seasonal modulation (Fig. 3e). AE activity peaks during the southeast monsoon (JJA), whereas CE occurrence reaches a maximum during the northwest monsoon (DJF). During CE peaks, eddies are broadly distributed across both northern and southern Indonesian seas, while AE maxima are concentrated primarily in the northern sector (Fig. 3f). This seasonal asymmetry reflects the strong modulation of mesoscale activity by monsoonal wind forcing and associated circulation changes.

Overall, the spatial structure, scale distribution, and seasonal variability of AEs and CEs demonstrate the widespread presence of dynamically coherent mesoscale eddies throughout the MC. These eddies contribute significantly to regional energy redistribution and mediate mass and property exchanges between adjacent basins, with implications for biological productivity and large-scale circulation^47–49^.

### Spatiotemporal distribution of eddy currents detected by the WA-OW method

**Fig. 4:**
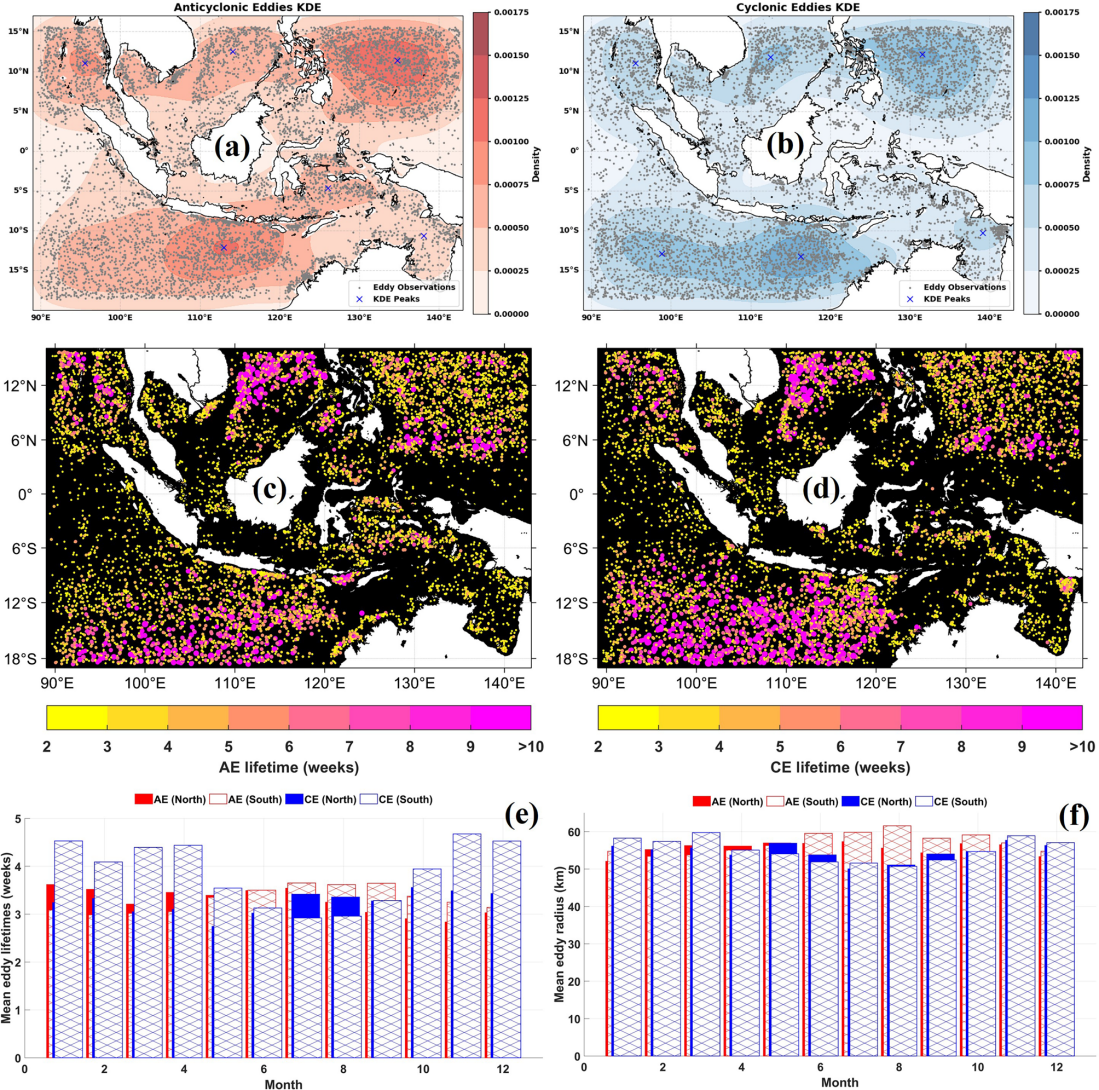
Spatial and temporal variability of eddies in the Maritime Continent (MC) from 1 January 1993 to 31 December 2022. (**a**) anticyclonic eddies (AEs); (**b**) cyclonic eddies (CEs) occurrence; spatial distribution of (**c**) AE and (**d**) CE lifetimes; monthly mean (**e**) lifetimes and (**f**) radius across hemispheres. Maps in (a–b) were created using Python (version 3.12.12) in Google Colab with Seaborn v0.13.2 (https://seaborn.pydata.org/) and Matplotlib v3.10.0; maps in (c, d) were created in MATLAB R2023b using M_Map (Pawlowicz, 2020, version 1.4 m, www.eoas.ubc.ca/~rich/map.html); panels (e–f) were generated in MATLAB R2023b.

Spatial variability is a defining feature of eddy activity in the MC. The distribution of AEs and CEs, along with their monthly variability across NH and SH, is shown in Fig. 4. AE hotspots occur primarily in the eastern South Java Sea and Northwest Pacific Ocean (Fig. 4a), while the eastern South Java Sea serves as a concurrent hotspot for CE activity (Fig. 4b). The Java Sea, Malacca Strait, and Celebes Sea consistently exhibit low eddy activity for both AE and CE, likely because of shallow bathymetry and weak geostrophic currents. The concentration of AEs in interior basins and CEs along boundary regions suggests distinct generation pathways shaped by basin geometry and dominant current systems^50^.

Spatial patterns in eddy lifetime (Fig. 4c, d) reveal heterogeneous dynamical regimes. Longer-lived eddies occur along the Northwest Pacific Ocean, where the variability of the Kuroshio Current modulates regional fields^51^. Offshore basins support longer lifetimes because interactions with topography, tidal mixing, and boundary currents are weak. In contrast, eddies in shallow and semi-enclosed seas dissipate rapidly. Regions such as the South Java Sea and Banda Sea exhibit accelerated decay due to lateral friction and enhanced coastal energy loss^52^.

Monthly eddy lifetimes (Fig. 4e) are dominated by durations of approximately three weeks, consistent with earlier studies^6,12^, which found that most Indonesian eddies persist for 21 to 28 days. These short-lived structures reflect seasonal current reversals, shelf–basin interactions, and background instabilities^53,54^.

Eddy radii vary with basin size (Fig. 4f), background current strength, coastline proximity, and topographic structure^55^. South of Java Sea, radii variability is particularly complex because of narrow topographic constraints and strong seasonal currents^56^. Across the archipelago, radius variability aligns with deformation-radius estimates, confirming the mesoscale nature of most detected eddies^57^.

**Fig. 5:**
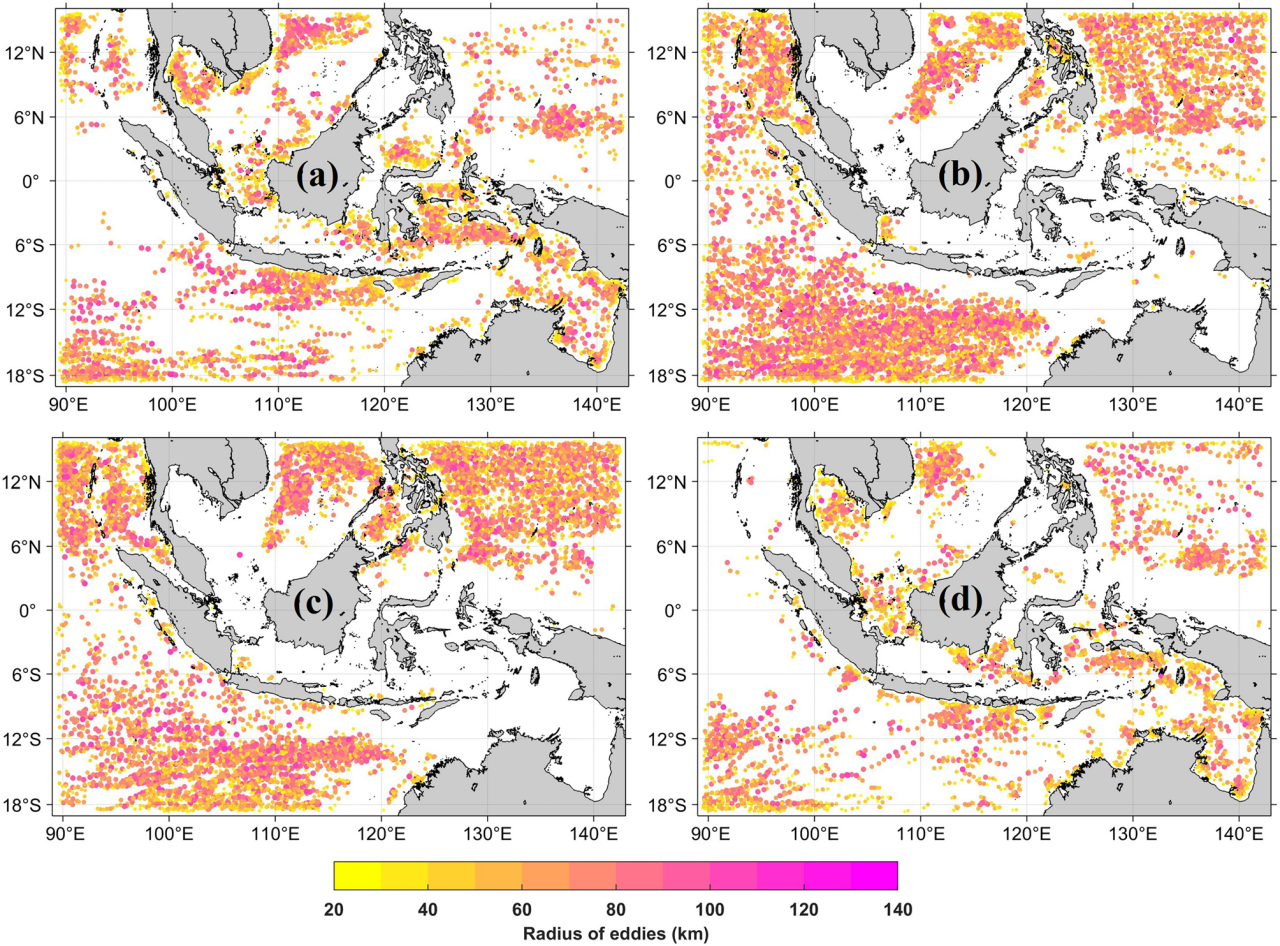
Seasonal distribution of eddy centers with radii of 20 to 140 km based on Winding Angle-Okubo Weiss (WA–OW) detection. (**a**) anticyclonic eddies (AEs) during DJF; (**b**) cyclonic eddies (CEs) during DJF; (**c**) AEs during JJA; (**d**) CEs during JJA. All maps were created in MATLAB R2023b using M_Map (Pawlowicz, 2020, version 1.4 m, www.eoas.ubc.ca/~rich/map.html*).*

Seasonal modulation of eddy characteristics is evident from the spatial distribution of eddy centers during DJF and JJA (Fig. 5). Eddy centers with radii of 20 to 140 km show enhanced AE occurrence during the southeast monsoon (Fig. 5a, c), whereas CE occurrence increases during the northwest monsoon (Fig. 5b, d). Seasonal differences extend to radius, vorticity, and lifetime, with CE showing enhanced values during DJF and AE during JJA. These patterns demonstrate the influence of monsoonal wind forcing and ocean–atmosphere interactions on eddy formation and evolution, consistent with wind-driven, baroclinic, and topographic controls on mesoscale variability in the region^35,58^.

**Fig. 6:**
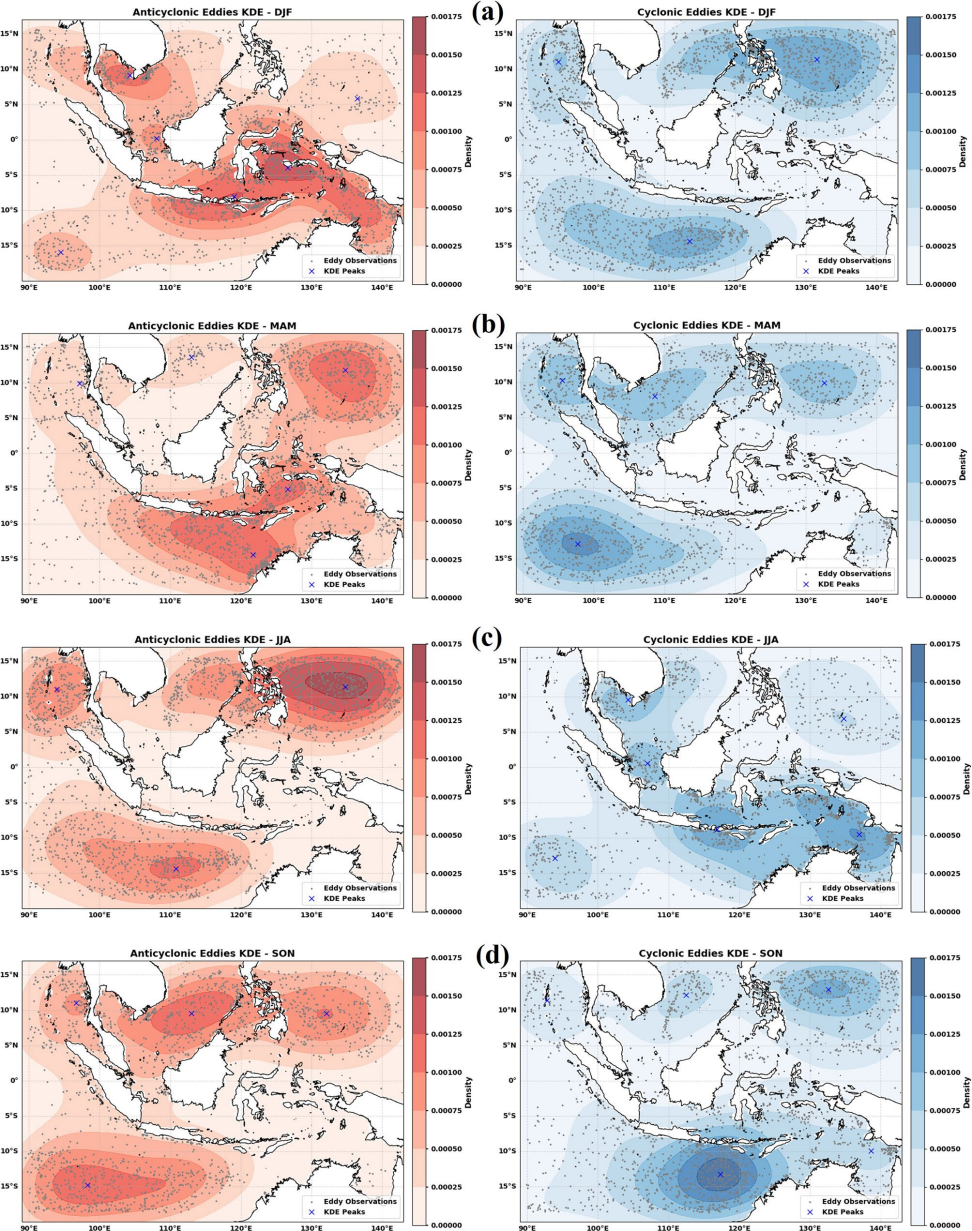
Seasonal spatial distribution and kernel density estimation (KDE) of anticyclonic eddies (AEs, left panels) and cyclonic eddies (CEs, right panels) in semi-enclosed basins of the Maritime Continent from 1 January 1993 to 31 December 2022. Black dots represent individual eddy observations derived from the WA–OW detection framework, while blue crosses denote KDE maxima (density peaks), indicating persistent eddy hotspots. Seasonal panels correspond to (**a**) December–February (DJF), (**b**) March–May (MAM), (**c**) June–August (JJA), and (**d**) September–November (SON). All maps were generated using Python 3.12.12 in Google Colab with Seaborn v0.13.2 (https://seaborn.pydata.org) and Matplotlib v3.10.0.

Regional heterogeneity is further illustrated in Fig. 6, which shows the distribution of AE and CE occurrences across semi-enclosed basins. The Banda Sea, Celebes Sea, and Savu Sea show concentrated eddy activity where internal dynamics interact with wind stress and topography (Kida and Wijffels, 2012).

Monsoonal wind patterns strongly modulate eddy generation and intensity. Seasonal wind reversal alters surface circulation and modifies EKE, particularly during the northwestern monsoon when intensified winds increase surface energy input. Seasonal differences in eddy lifetime reflect these changing conditions. Eddies that form during warmer months may intensify rapidly but weaken under seasonal cooling, whereas eddies forming during cooler months experience slower decay^59^. These patterns demonstrate the tight coupling between atmospheric forcing and oceanic response.

The ITF transports warm, low-salinity Pacific waters through the Makassar Strait, Flores Sea, and Banda Sea toward the Indian Ocean. This sustained transport enhances horizontal shear and density gradients that promote baroclinic and barotropic instabilities, generating mesoscale eddies along the ITF pathway^60^. Seasonal variations in the ITF regulate eddy behavior, with enhanced AE activity during DJF and increased CE occurrence during JJA in key basins (Fig. 6). Alternating monsoon winds shape Ekman transport, steepen meridional sea level gradients, and intensify horizontal shear^12^. Monsoonal forcing also alters thermocline water volume, which thickens during the southeast monsoon^61^. Because ITF transport covaries with thermocline volume, stronger throughflow during JJA enhances eddy-related volume transport into the Indian Ocean^35^.

At the ITF exit passages, eddy activity displays clear seasonal phasing. Peak transport during JJA deepens the thermocline, enhancing pycnocline variability and promoting baroclinic instability. This mechanism explains the stronger eddy intensity during JJA, consistent with earlier ITF–eddy coupling studies^62^. The ITF therefore acts both as a generator of eddies through shear and stratification changes and as a regulator of their seasonal intensity.

Mesoscale eddies in the MC mediate basin-scale ocean–climate interactions through their coupling with the ITF, monsoonal forcing, and air–sea exchange. Many strong eddy centers align with primary ITF pathways, including the Makassar Strait, Banda Sea, and Halmahera Sea. Eddies modify ITF transport by redistributing momentum, heat, and tracers both vertically and laterally. Seasonal differences in wind forcing shape the strength of eddy–mean flow interactions. During boreal winter, enhanced westerlies strengthen eddies that can deflect ITF flow along topographic boundaries, while during boreal summer, weaker winds allow geostrophic currents to dominate^58^. These processes show that eddies function as an intermediate pathway through which atmospheric forcing modifies interior ocean variability.

Changes in eddy intensity and polarity influence mixed-layer stratification, SST, biological productivity, and surface heat fluxes^63^. Strong winter eddies enhance nutrient fluxes through upwelling, while warm-core structures suppress entrainment and retain surface heat. Spatial heterogeneity in SST can modify surface winds, latent heat flux, and boundary layer stability^12^. These results show that mesoscale eddies in the MC play a dynamic role in shaping ITF variability, regional monsoon expression, and air–sea coupling. Future work involving surface heat flux diagnostics and transport budgets may better quantify these interactions and their climatic significance^64^.

### Kinematic parameters of eddy current circulation

**Fig. 7:**
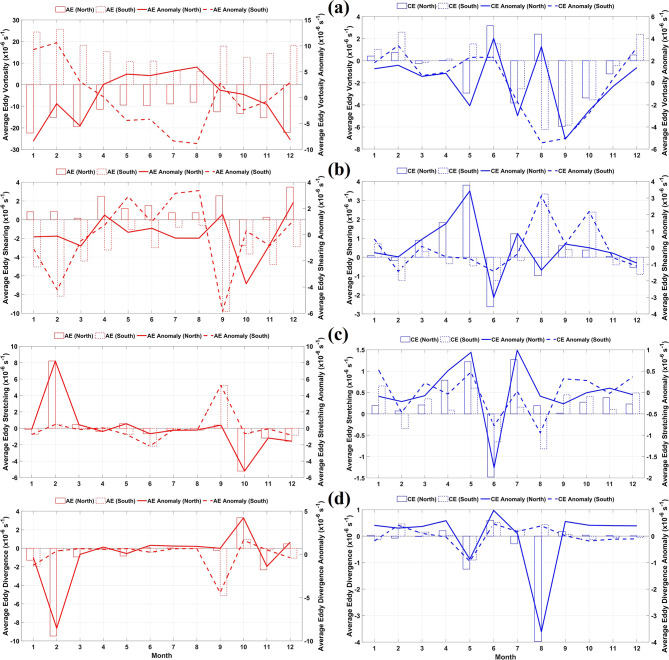
Monthly variation (**a**) vorticity, (**b**) shearing, (**c**) stretching, and (**d**) divergence of anticyclonic eddy (AE) and cyclonic eddy (CE) for the northern and southern part of the Maritime Continent (MC) from 1993 to 2022. All panels were generated in MATLAB R2023b.

Kinematic parameters provide mechanistic insight into eddy evolution across monsoonal phases. Figure 7 shows the strong seasonal variability in vorticity, shearing, stretching, and divergence. In the SH, AE vorticity peaks during the northwestern monsoon in February and declines through MAM to its lowest values during the southeastern monsoon (Fig. 7a). In the NH, AE vorticity reaches its maximum in January and declines sharply in February. CE vorticity is generally weaker and fluctuates in sign, likely because of complex background flows and topographic modulation. In the NH, CE attains peak intensity during the southeastern monsoon in August, while in the SH, CE varies distinctively between May and August. These contrasting AE and CE signatures reflect differing generation mechanisms and sensitivities to regional forcing.

Deformation fields also exhibit strong seasonal patterns. Shearing deformation is particularly pronounced in NH AE, increasing from a minimum in August to a peak in September, indicating enhanced lateral shear associated with current reversals (Fig. 7b). This contributes to deviations from circular eddy structure. CE shearing is weaker and shows slight variation. Stretching deformation exhibits irregular transitions in both AE and CE, with CE experiencing pronounced stretching in February and September and contraction in October (Fig. 7c). These patterns indicate non-linear interactions between mesoscale eddies and transient jets^65,66^.

Divergence patterns highlight vertical exchanges within the water column (Fig. 7d). Northern AE in February exhibits strong negative divergence of approximately 9.5 × 10^−^⁶ s^−1^, suggesting downwelling at the core and potential upwelling at the periphery. CE divergence is relatively stable, although northern CE in August shows contraction near 3.5 × 10⁻⁶ s^−1^. These divergence–convergence structures modulate vertical stratification and nutrient entrainment, influencing biological productivity (Cabrera et al., 2022). This reflects transitions between deformation-dominant and vorticity-dominant states, highlighting brief episodes of dynamical resonance when external forcing aligns with optimal eddy growth conditions.

**Fig. 8:**
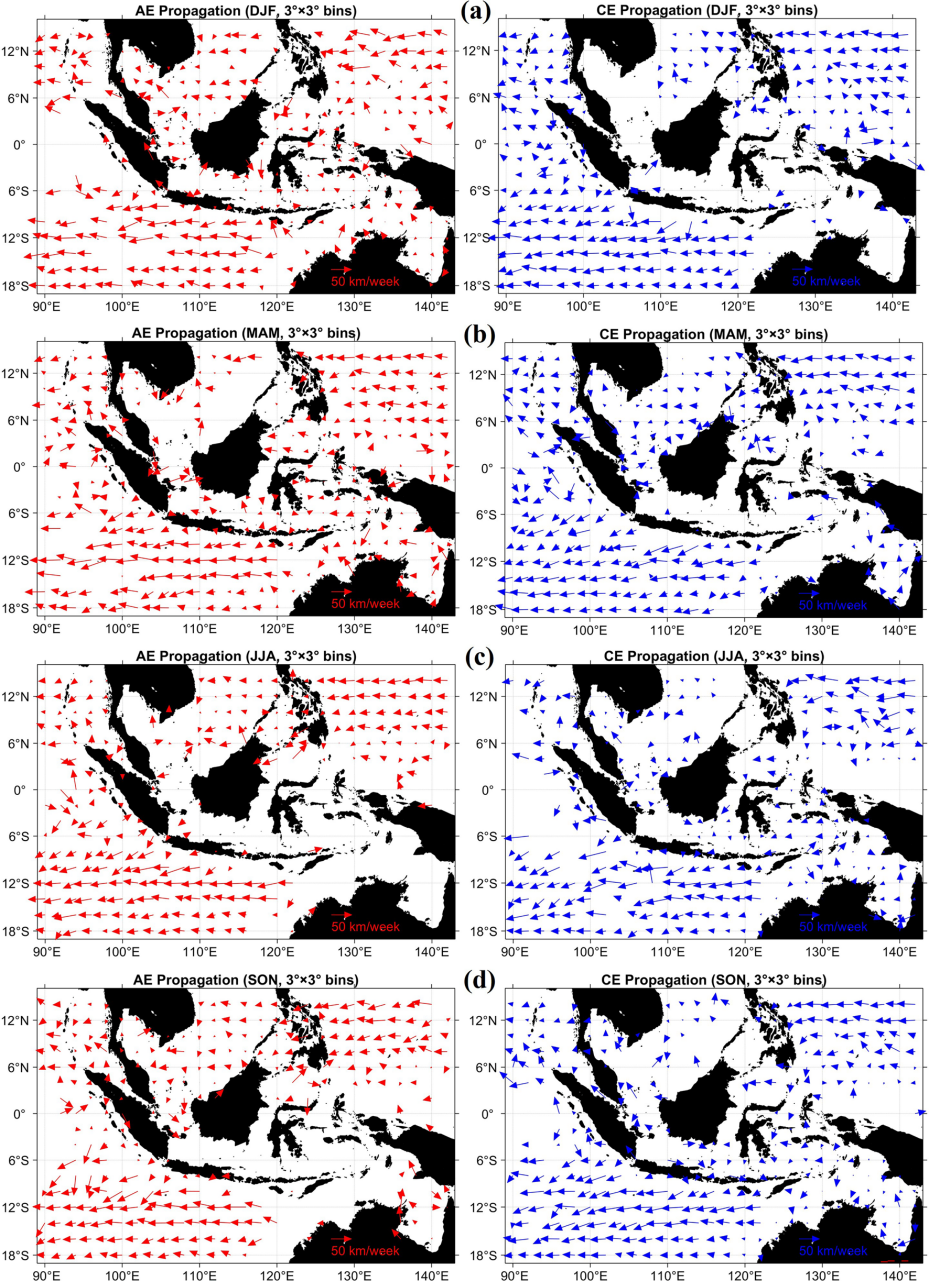
Seasonal propagation velocity and direction of anticyclonic eddies (AE) and cyclonic eddies (CE) in the Indonesian seas. Propagation patterns are shown for (**a**) December–February (DJF), (**b**) March–May (MAM), (**c**) June–August (JJA), and (**d**) September–November (SON). Propagation velocity was calculated as the lifetime-mean displacement between the initial and final eddy centers divided by eddy lifetime and converted to km week⁻^1^. Velocities were averaged within 3° × 3° spatial bins. Arrows indicate the mean propagation direction, and arrow length is proportional to propagation speed. Red arrows represent AEs and blue arrows represent CEs. All maps were created in MATLAB R2023b using M_Map (Pawlowicz, 2020, version 1.4 m, www.eoas.ubc.ca/~rich/map.html).

Seasonal propagation characteristics exhibit pronounced regional contrasts (Fig. 8). In the Sulu Sea, AEs propagate predominantly southeastward with typical speeds of approximately 5–20 km week^−1^, whereas CEs move more slowly, generally at 2–10 km week^−1^. In the Sulawesi and Maluku Seas, eddy trajectories are primarily westward to southwestward, consistent with Makassar Strait intrusion and regional basin geometry. In the Banda Sea, CE propagation becomes predominantly eastward during austral summer (DJF), while AEs tend to migrate southeastward.

Propagation velocity was estimated from the displacement between the initial and final eddy centers divided by eddy lifetime and converted to km week^−1^. These velocities were then spatially averaged within 3° × 3° bins to determine the dominant seasonal propagation vectors. In Fig. 8, arrow direction indicates the mean propagation direction within each bin, and arrow length is proportional to propagation speed. Red arrows denote AEs and blue arrows denote CEs.

The observed spatial and seasonal variability reflects the combined influence of background circulation, monsoonal wind forcing, and basin-scale bathymetric constraints, underscoring the dynamically heterogeneous nature of this semi-enclosed MC basin. Consistent with previous studies, eddy activity intensifies during boreal winter in the Banda and Halmahera Seas, in agreement with the wind-driven mechanisms reported by Hao et al.^12^. The application of the $\beta$-plane correction further enhances the physical consistency of vorticity and geostrophic velocity fields, particularly in narrow passages and semi-enclosed basins, leading to a more robust representation of eddy structure and variability.

### Schematic of eddy circulation in semi-enclosed basins in the Maritime Continent

To clarify the dynamic processes controlling the seasonal variability of mesoscale eddies, schematic diagrams were developed for key semi-enclosed basins across the Indonesian Seas. Peak months differ by region: March and September in the South Java Sea, January and September in the Celebes Sea, February and August in the Flores Sea, January and August in the Savu Sea, March and August in the Banda Sea, and March and August in the Arafura Sea. These patterns reflect the combined influence of monsoonal wind stress, ITF variability, and bathymetric steering.

During the NWM season (December to March), westerly wind bursts enhance northward Ekman transport and steepen meridional sea level gradients, strengthening the western ITF branch and increasing horizontal shear. This promotes CE formation in western basins and AE development in the east. During the SEM season (July to September), accelerated flow past major islands generates positive vorticity, producing CEs on upstream sides and AEs downstream. In the Arafura Sea, seasonal peaks arise from the combined effects of regional wind forcing and shallow, topographically constrained geometry.

- South Java Sea.

**Fig. 9:**
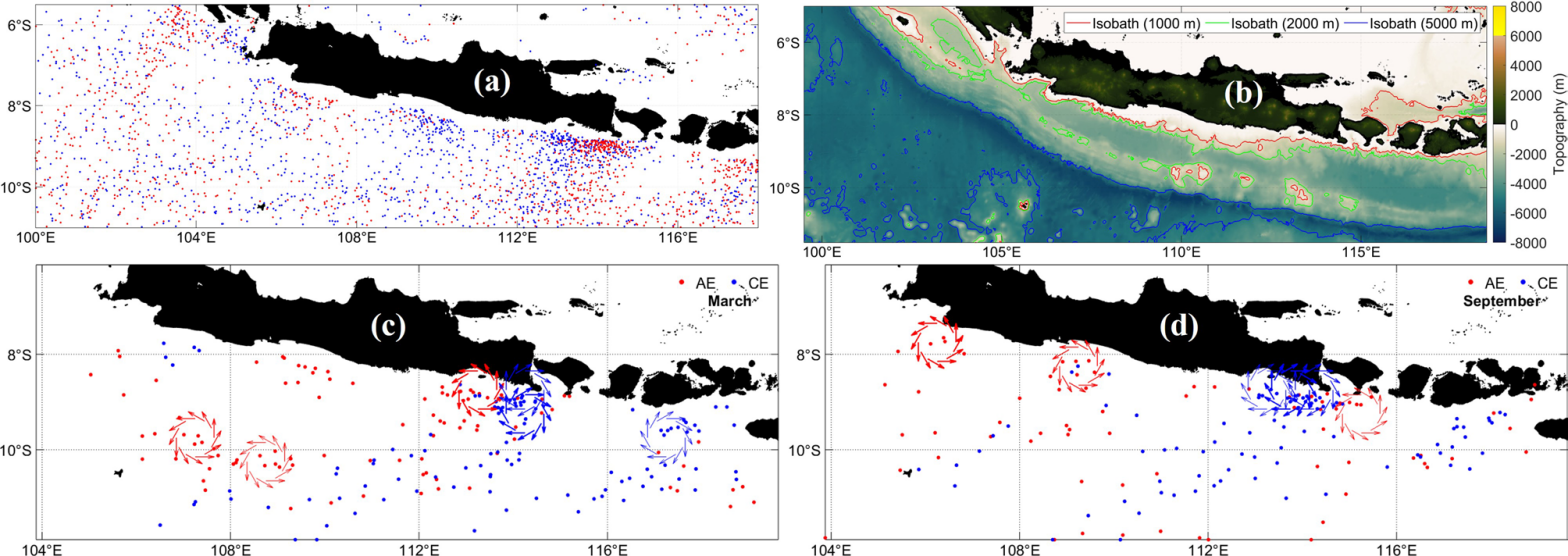
(**a**) Distribution of anticyclonic eddies (red) and cyclonic eddies (blue) at South Java Sea from 1993 to 2022. (**b**) Bathymetry that provides boundary constraints for eddy generation. (**c**) and (**d**) Schematics of AE and CE formation in March and September illustrate seasonal eddy responses to wind forcing and coastline interaction during the northwestern and southeastern monsoons. All maps were created in MATLAB R2023b using M_Map (Pawlowicz, 2020, version 1.4 m, www.eoas.ubc.ca/~rich/map.html).

Eddy activity in the South Java Sea shows strong seasonal modulation, which is illustrated in Fig. 9a, b display the spatial distribution of AEs and CEs and the bathymetry that constrains eddy formation. Figure 9c, d present the March and September schematics that depict eddy responses to monsoon-driven circulation. Eddies form along with sharp SLA gradients that reflect the linkage between surface pressure fields and mesoscale circulation. March eddies are associated with west monsoon forcing, whereas September eddies develop under strong northwestward flow during the east monsoon^67^. Interactions among the South Java Current, the South Equatorial Current, and ITF outflow enhance horizontal shear and support baroclinic and barotropic instabilities^62^. Variability in ITF transport through Lombok, Ombai, and Timor Straits influences eddy formation^68^. Eddies frequently propagate along the shelf break and may turn southward into the Indian Ocean^70^, reflecting the combined effects of monsoon forcing, boundary currents, and coastal geometry.

b. Celebes Sea.

**Fig. 10:**
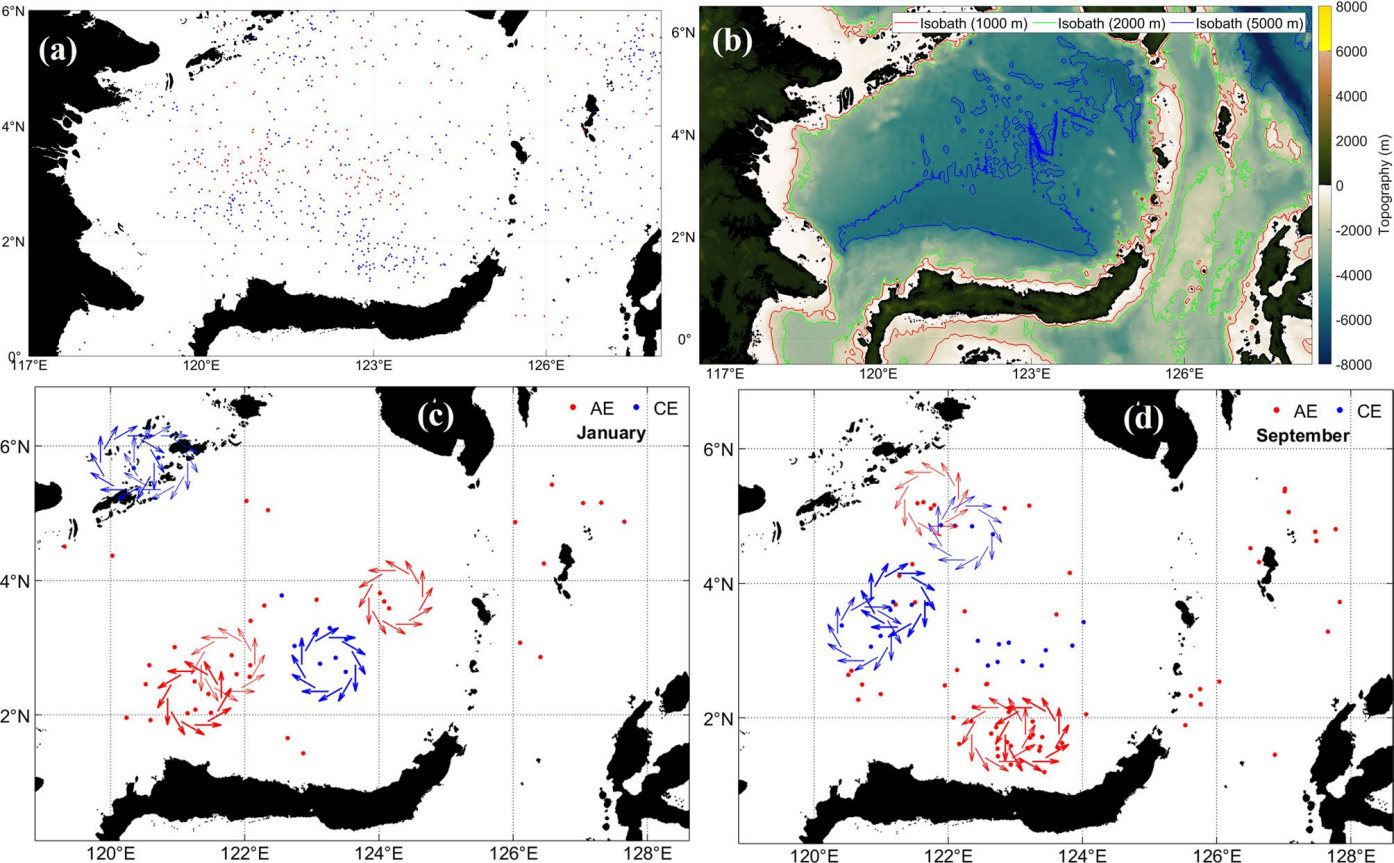
(**a**) Distribution of anticyclonic eddy (red) and cyclonic eddy (blue) at Celebes Sea from 1993 to 2022. (**b**) Bathymetry that constrains eddy generation. (**c**) and (**d**) Schematics for January and September illustrate seasonal wind-driven circulation and its modulation of eddy intensity and type. All maps were created in MATLAB R2023b using M_Map (Pawlowicz, 2020, version 1.4 m, www.eoas.ubc.ca/~rich/map.html).

The Celebes Sea hosts a dense eddy population shaped by strong SLA gradients and complex bathymetry, as shown in Fig. 10. Figure 10a displays AE and CE distribution, Fig. 10b shows bathymetry, and Fig. 10c–d present January and September schematics illustrating seasonal eddy formation. AEs occur in regions of elevated SLA, whereas CEs align with negative SLA. Enhanced geostrophic currents near steep fronts, particularly around 123°E, indicate regions of baroclinic instability. Eddy activity follows the 2000 m isobaths, highlighting the role of topography. ITF inflow, contributions from the Sulu Sea, and return flows associated with retroflection or wind interactions modulate eddy intensity^35^. Seasonal variability is strong, with January showing peak eddy occurrence during the northeast monsoon when northeasterly winds intensify shear^71^.

Eddies play important biogeochemical roles. AE laterally traps and redistributes warm water, affecting stratification, while CE promotes upwelling of nutrient-rich waters that enhance productivity^72^. Eddy interactions with steep topography regulate energy dissipation and influence regional circulation pathways, with potential impacts on monsoon variability and ENSO-linked climate signals^73^.

c. Flores Sea.

**Fig. 11:**
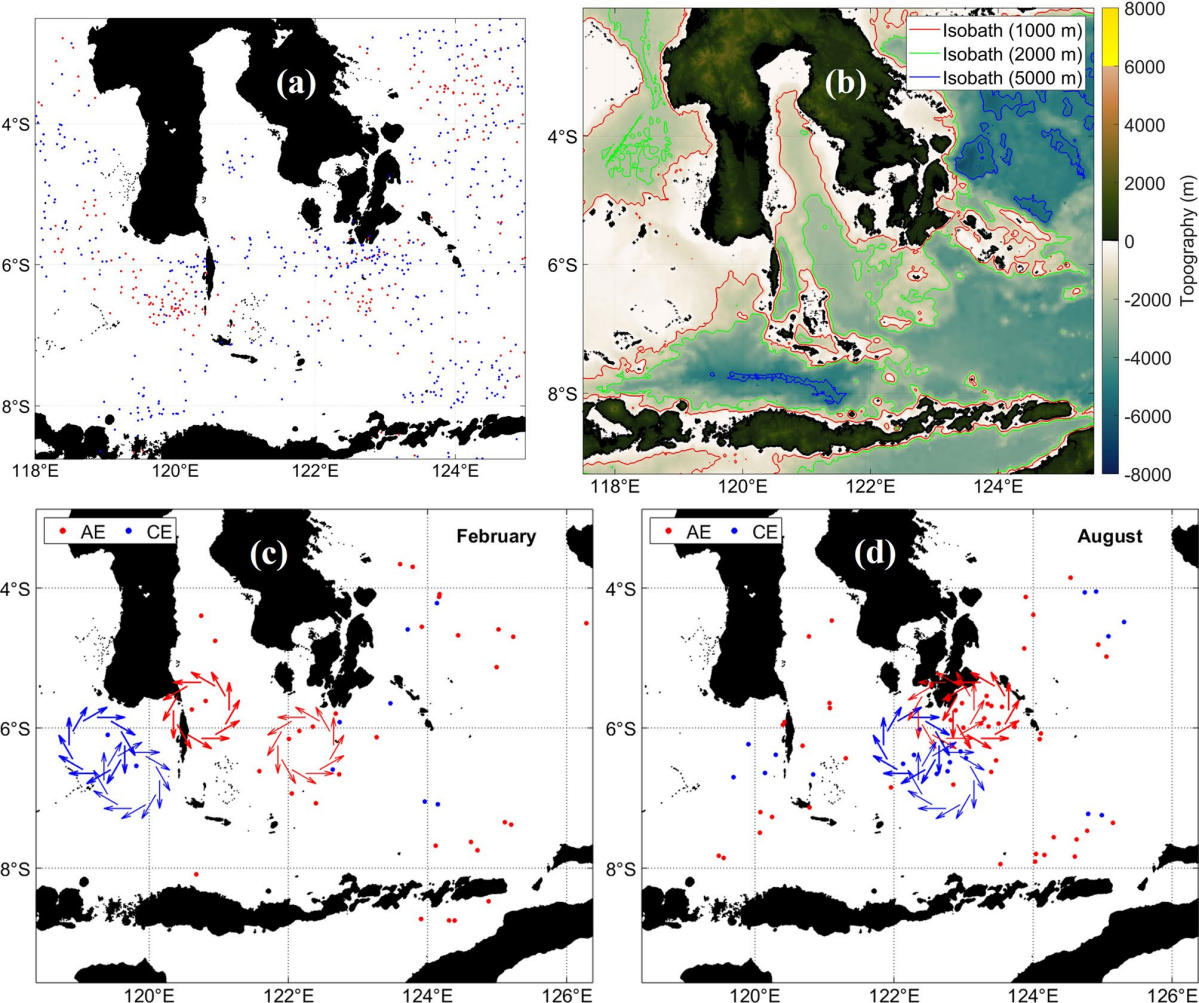
(**A**) Distribution of anticyclonic eddy (red) and cyclonic eddy (blue) at Flores Sea﻿ from 1993 to 2022. (**b**) Bathymetry that forms boundary conditions for eddy generation. (**c**) and (**d**) Schematics for February and August illustrate monsoon-driven circulation and seasonal modulation of eddy formation. All maps were created in MATLAB R2023b using M_Map (Pawlowicz, 2020, version 1.4 m, www.eoas.ubc.ca/~rich/map.html).

The highly energetic mesoscale environment of the Flores Sea is captured in Fig. 11. Figure 11a shows AE and CE distributions, Fig. 11b presents the bathymetry that governs boundary constraints, and Fig. 11c–d illustrate February and August seasonal schematics. Westward flow dominates south of 8°S, while northern regions exhibit variable currents shaped by island chains and topographic complexity. SLA distributions delineate AE and CE centers, and mesoscale activity clusters near constricted passages such as the Lombok Strait.

Eddy formation arises from instabilities in the ITF-dominated flow and from topographic steering, where islands and sills generate shear and potential vorticity gradients conducive to baroclinic and barotropic instabilities^74^. The heterogeneous distribution of eddies reflects localized forcing and bathymetric control. AEs with warm, saline cores contribute to the westward transport of heat and Pacific waters, influencing the ITF heat and freshwater budget^75^. CEs promote eddy-induced upwelling, creating productivity hotspots. These eddies also participate in the regional energy cascade, redistributing kinetic energy toward smaller scales where it is dissipated^76^. Their influence on SST and stratification underscores their importance for air-sea interactions and Indo-Pacific climate dynamics.

d. Savu Sea.

**Fig. 12:**
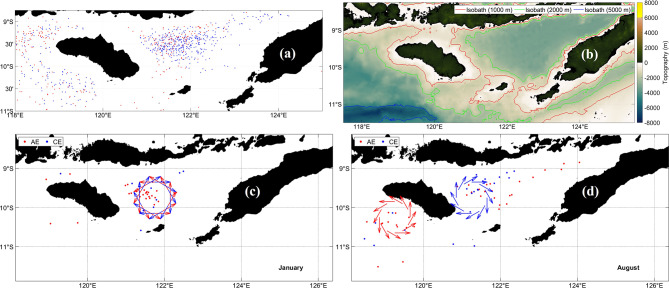
(**a**) Distribution of anticyclonic eddy (red) and cyclonic eddy (blue) at Savu Sea﻿ from 1993 to 2022. (**b**) Bathymetry that constrains eddy generation. (**c**) and (**d**) Schematics for January and August that represent eddy activity peaks driven by monsoonal circulation and boundary current variability. All maps were created in MATLAB R2023b using M_Map (Pawlowicz, 2020, version 1.4 m, www.eoas.ubc.ca/~rich/map.html).

Energetic mesoscale variability in the Savu Sea is displayed in Fig. 12, where Fig. 12a maps AE and CE distributions, Fig. 12b shows bathymetry, and Fig. 12c–d depict January and August schematics of peak eddy activity. Westward flow dominates south of 10°S, while northern circulation is shaped by bathymetric features and island passages. SLA highs correspond to AEs and SLA lows to CEs. Eddy clusters occur near constricted passages that enhance shear.

Instabilities in ITF flow and strong topographic steering generate both AEs and CEs. AEs transport heat and Pacific waters westward, while CEs drive nutrient-rich upwelling that supports productivity^77,78^. These eddies regulate the regional energy cascade and modulate SST, stratification, and air-sea coupling^65^. Their seasonal variability reflects monsoonal wind forcing and changes in boundary currents.

e. Banda Sea.

**Fig. 13:**
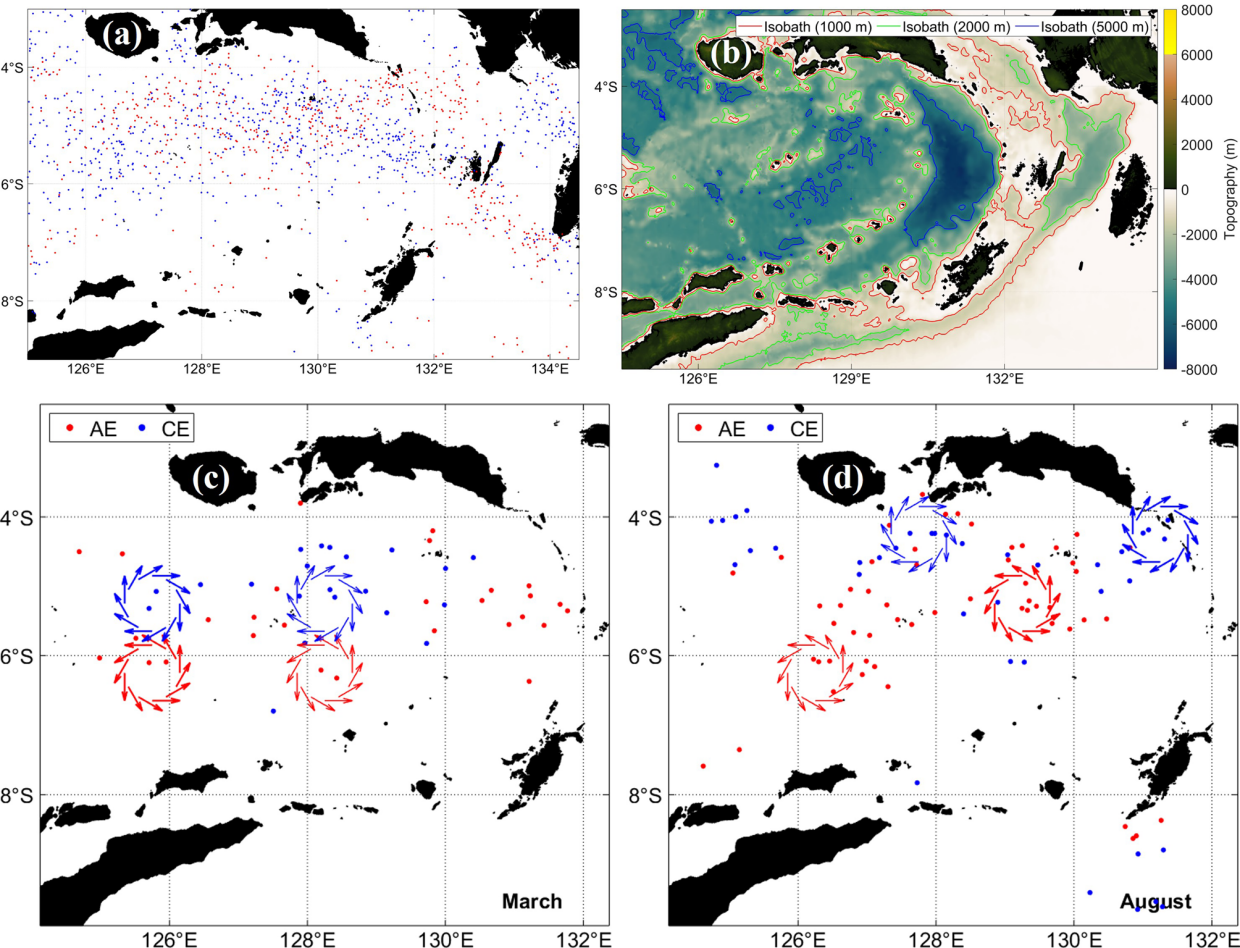
(**a**) Distribution of anticyclonic eddy (red) and cyclonic eddy (blue) at Banda Sea﻿ from 1993 to 2022. (**b**) Bathymetry that constrains eddy generation. (**c**) and (**d**) Schematics for March and August showing distinct eastern and western eddy types formed by wind stress curl and ITF–topography interactions. All maps were created in MATLAB R2023b using M_Map (Pawlowicz, 2020, version 1.4 m, www.eoas.ubc.ca/~rich/map.html).

Seasonal eddy formation in the Banda Sea is illustrated in Fig. 13. Figure 13a shows the spatial eddy distribution, Fig. 13b shows bathymetry, and Fig. 13c–d present March and August schematics that show contrasting eastern and western eddy types. During the NWM, westerly winds strengthen northward Ekman transport, steepen meridional sea level gradients, and increase horizontal shear^58^. These conditions promote CEs in the west and AEs in the east. During the SEM, intensified flow past Buru Island generates positive vorticity that again produces CEs upstream and AEs downstream.

Ekman-induced downwelling during NWM promotes AEs^12^, while SEM upwelling supports CEs^62^. Eddy variability is shaped by monsoon winds, ITF pathways, and steep MC topography^79^, explaining the distinct seasonal phasing of eddy types.

f. Arafura Sea.

**Fig. 14:**
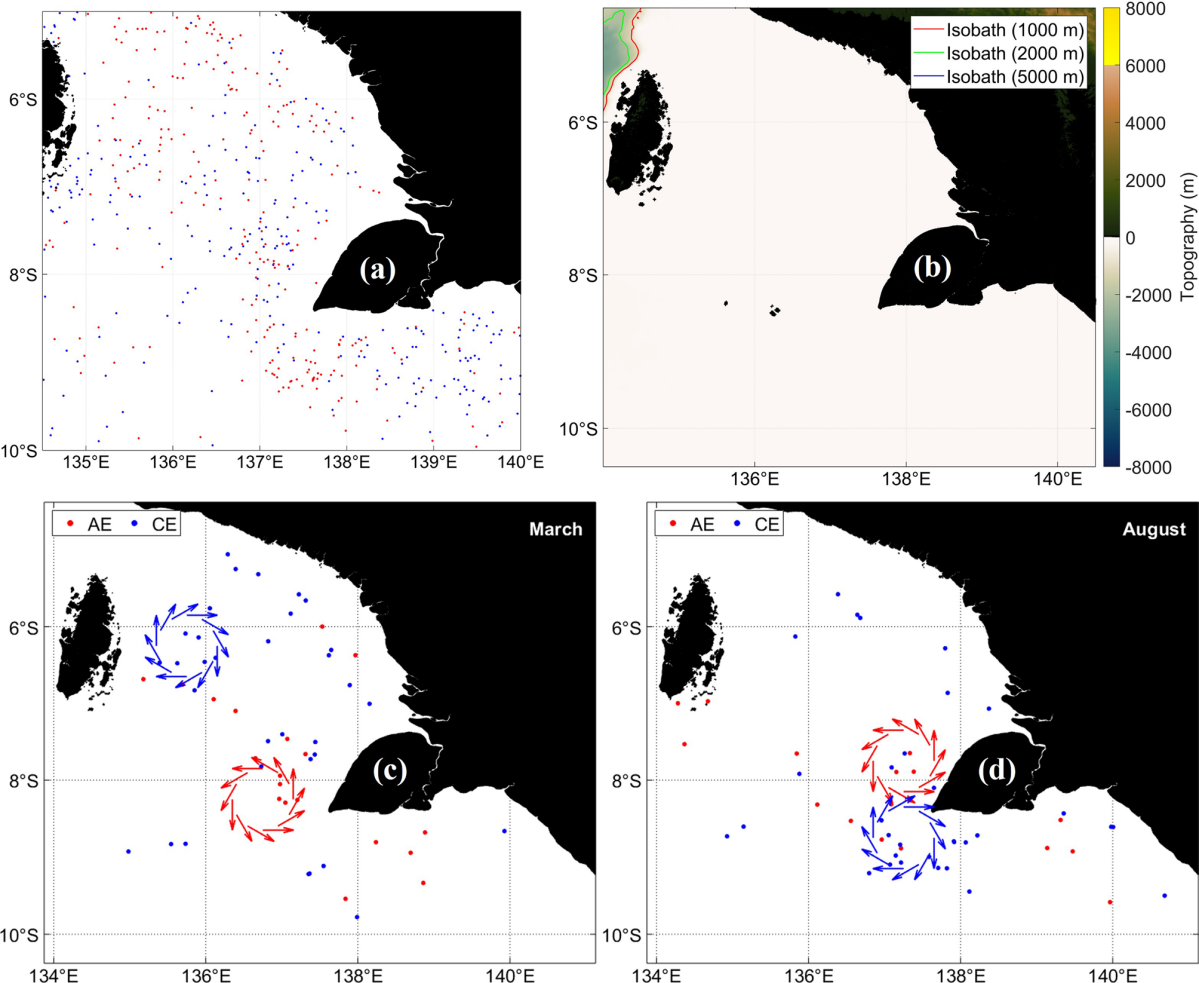
(**a**) Distribution of anticyclonic eddy (red) and cyclonic eddy (blue) at Arafura Sea﻿ from 1993 to 2022. (**b**) Bathymetry that constrains eddy generation. (**c**) and (**d**) Schematics for March and August showing seasonal changes in wind forcing and its interaction with shallow bathymetry and shelf geometry. All maps were created in MATLAB R2023b using M_Map (Pawlowicz, 2020, version 1.4 m, www.eoas.ubc.ca/~rich/map.html).

The strong mesoscale activity of the Arafura Sea is captured in Fig. 14. Figure 14a shows AE and CE distributions, Fig. 14b shows bathymetry, and Fig. 14c–d depict March and August schematics that illustrate the influence of seasonal wind forcing, shallow bathymetry, and shelf geometry. A persistent southward current between 136.5°E and 137.5°E separates predominantly AEs to the west from more variable CEs to the east. Eddy clustering along SLA fronts reflects baroclinic instability generated by shear zones and bathymetric contrasts.

Eddy formation results from interactions among regional currents, monsoonal winds, and topographic steering^23^. Seasonal SLA anomalies, with positive values in March and negative in August, demonstrate monsoon-driven adjustments that enhance instability^67,80,81^. Wind-driven divergence and eddy-induced vertical motion dominate the seasonal signal^82^. Larger and longer-lived eddies develop during the second monsoon transition in September and October, when winds intensify and oceanic instability increases^83^.

g. South China Sea.

Mesoscale eddy formation in the SCS is strongly influenced by monsoon-driven wind forcing, basin-scale baroclinic structure, and complex topography. During the southwest monsoon, positive wind stress curl off central Vietnam induces Ekman suction and thermocline shoaling, favoring the development of CEs through enhanced upper-ocean divergence[^85^]. In contrast, the northeast monsoon strengthens basin-scale circulation and modifies regional vorticity forcing, contributing to AEs generation in boundary regions^84^. The seasonal alternation between CE and AE dominance reflects monsoon-modulated wind stress curl and the growth of baroclinic instability within the stratified basin circulation^86^.

h. Philippine Sea

The formation of eddies in the Philippine Sea is strongly influenced by the interaction between large-scale ocean currents and regional atmospheric forcing^87^. A key mechanism is the bifurcation of the North Equatorial Current (NEC) upon reaching the Philippine coast, where part of the flow is directed northward into the Kuroshio Current, the major western boundary current of the Pacific, while the other branch turns southward into the Mindanao Current^88^. The latitude of this bifurcation is not fixed but varies seasonally and interannually under the influence of monsoon winds and ENSO-related variability, modulating horizontal velocity shear and baroclinic instability that promote mesoscale eddy generation^89^. In addition, the Kuroshio Current often meanders as it flows northward along the Philippine slope, and these meanders interact with the regional bathymetry to enhance vortex shedding and eddy growth^90^. The complex submarine topography in the basin, including trenches and ridges, deflects current pathways and contributes to localized vorticity generation. Furthermore, wind stress curl associated with monsoonal circulation drives Ekman pumping, which enhances vertical motion and modulates upper-ocean potential vorticity, favoring the development of both CEs and AEs. Together, these processes make the Philippine Sea a highly dynamic region where eddy activity reflects the combined effects of current bifurcation, western boundary current variability, atmospheric forcing, and topographic control.

### Intertropical convergence zone (ITCZ) influences eddy variability

**Fig. 15:**
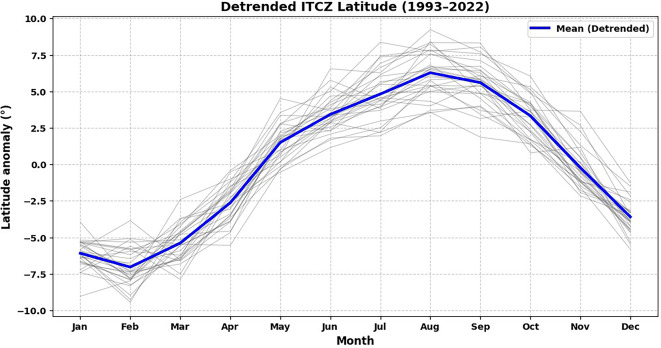
Mean detrended ITCZ latitude during 1993–2022. The blue line shows the multi-year mean ITCZ latitude, while the grey lines represent the ITCZ latitude for each individual year. Panel created using Python 3.12.12 in Google Colab with Seaborn v0.13.2 (https://seaborn.pydata.org) and Matplotlib v3.10.0.

While the earlier analysis described basin‐wide seasonal variations in eddy activity, we now separate the statistics by hemisphere to investigate how eddy occurrence relates to the meridional migration of the ITCZ. The ITCZ is a fundamental feature of tropical atmospheric circulation, and its seasonal shifts are known to reorganize surface winds and air–sea momentum fluxes across the equator. To isolate interannual–seasonal variability and avoid spurious correlations arising from long‐term ITCZ displacement, the ITCZ latitude was detrended over the period 1993–2022 (Fig. 15). This approach allows the analysis to focus on physically meaningful short‐term ITCZ fluctuations that are more directly linked to hemispheric differences in CE and AE activity.

**Fig. 16:**
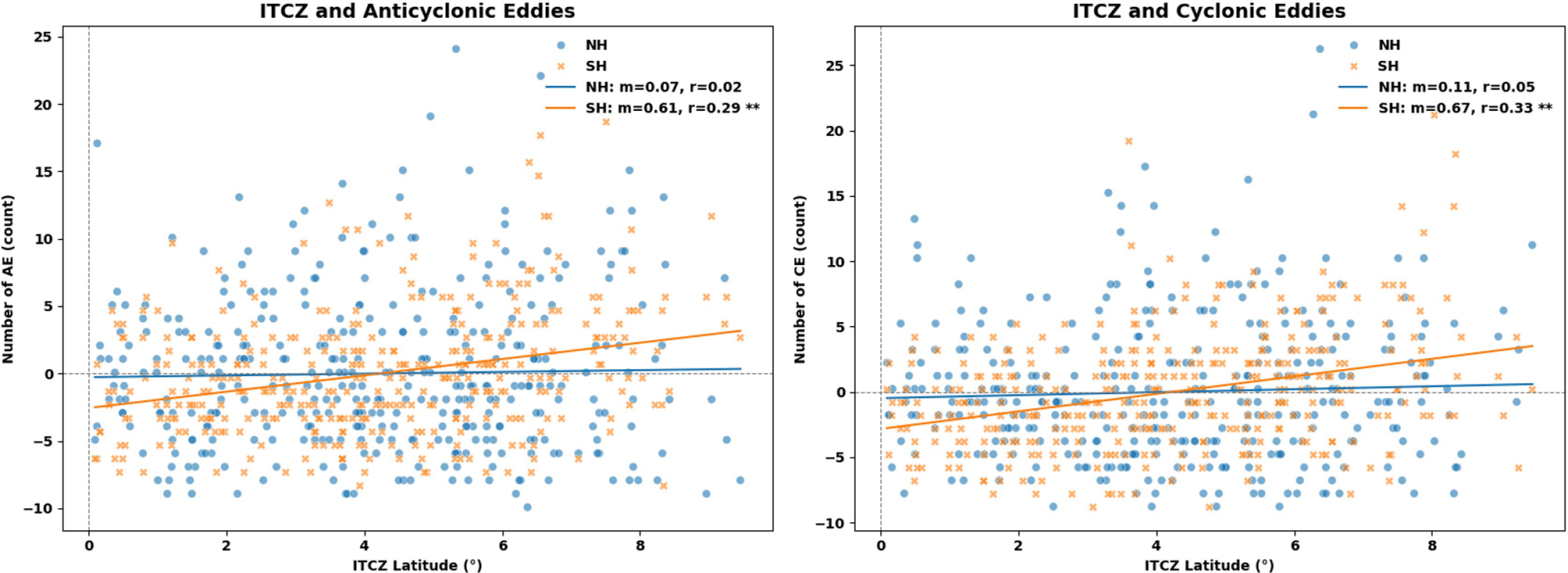
Linear regression between detrended ITCZ latitude and eddy counts. All panels were created using Python 3.12.12 in Google Colab with Seaborn v0.13.2 (https://seaborn.pydata.org) and Matplotlib v3.10.0.

From the correlation analysis between eddy occurrence and ITCZ latitude, CE exhibits a significant negative relationship with ITCZ latitude in the NH ($r$ = − 0.59) (Fig. 16), indicating that a northward displacement of the ITCZ is associated with reduced CE occurrence in the NH. In contrast, the SH shows a positive correlation ($r$ = + 0.29), suggesting that a more northward ITCZ is linked to enhanced CE activity in the SH. AE displays the opposite pattern, with a significant positive correlation in the NH ($r$ = + 0.54) and a significant negative correlation in the SH ($r$ = − 0.54).

Seasonal variations (Table 2) generally reflect the all-year pattern, with the seesaw-like response between hemispheres being most pronounced during MAM and SON, and relatively weaker during DJF. Overall, these results indicate that the latitudinal position of the ITCZ is statistically associated with variations in eddy activity, but the nature of the relationship differs between hemispheres. A northward ITCZ shift tends to suppress CE in the NH while enhancing it in the SH, whereas AE responds oppositely.

**Table 2:** Correlation between ITCZ latitude and the number of eddies.

Season	CE			AE		
	All area	NH	SH	All	NH	SH
All year	− 0.29**	− 0.59**	+ 0.29**	+ 0.10	+ 0.54**	− 0.54**
DJF	+ 0.00	+ 0.09	− 0.12	− 0.14	+ 0.10	− 0.30**
MAM	− 0.44**	− 0.42**	− 0.27**	+ 0.19	+ 0.34**	− 0.57**
JJA	+ 0.50**	− 0.02	+ 0.52**	+ 0.15	+ 0.11	+ 0.10
SON	+ 0.12	− 0.38**	− 0.52**	+ 0.40**	+ 0.44	+ 0.06

**) indicate significant statistically with p > 0.05.

Our results reveal a clear hemispheric asymmetry in how eddy activity responds to ITCZ displacement. CEs show a strong negative correlation with ITCZ latitude in the NH ($r = -0.59$), meaning that a northward ITCZ shift suppresses CE activity. In contrast, CEs in the SH increase during northward ITCZ excursions ($r = +0.29$). AEs respond in the opposite manner, with a positive correlation in the NH ($r = +0.54$) and a negative one in the SH (r = − 0.54). These hemispheric “seesaw” responses are most pronounced during MAM and SON, and somewhat weaker during DJF.

**Fig. 17:**
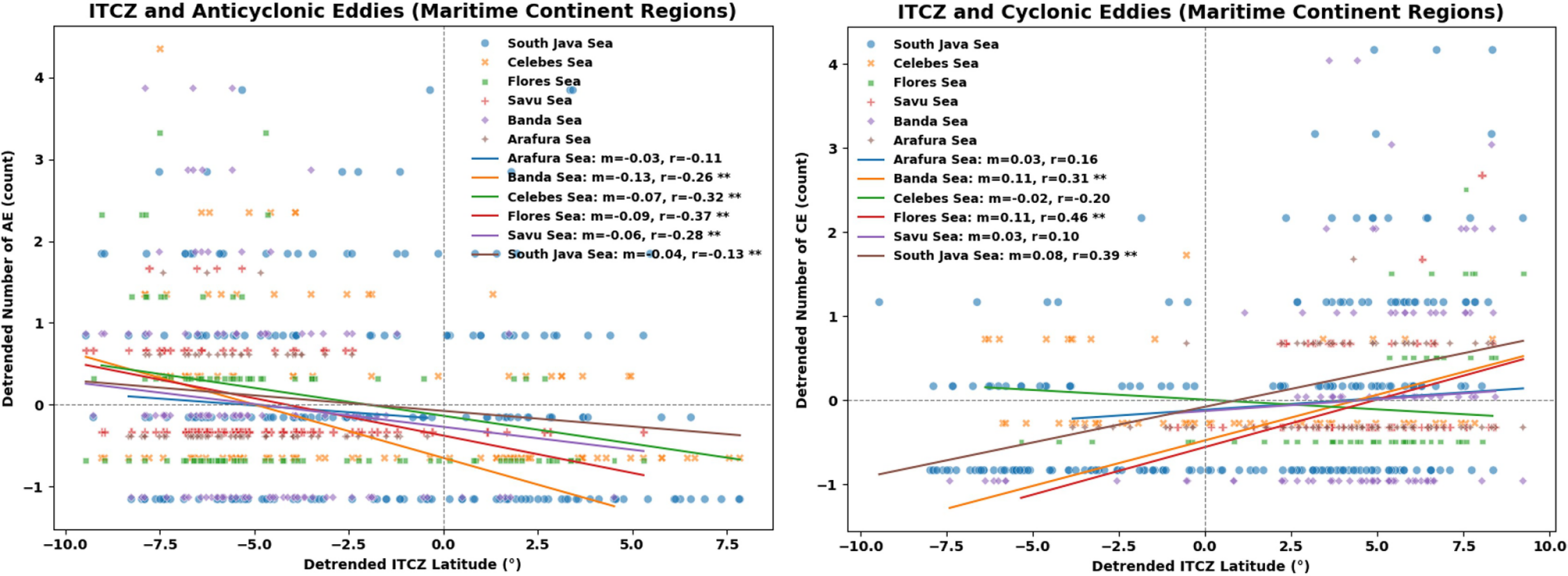
Same as 16 but for Maritime Continent (MC) Regions. All panels were created using Python 3.12.12 in Google Colab with Seaborn v0.13.2 (https://seaborn.pydata.org) and Matplotlib v3.10.0.

To further assess the spatial expression of ITCZ-related forcing within the MC basin, we extended the linear regression analysis to individual sub-regions, including the Arafura Sea, Banda Sea, Celebes Sea, Flores Sea, Savu Sea, and the South Java Sea (Fig. 17). This regional breakdown reveals that the eddy–ITCZ relationship is not uniform across the basin but instead exhibits pronounced geographic heterogeneity.

For CEs, significant correlations with ITCZ latitude emerge most clearly in the central Indonesian seas, with the strongest responses found in the Flores Sea (*r* = 0.46), the South Java Sea (*r* = 0.39), and the Banda Sea (*r* = 0.31). AEs show a complementary but distinct pattern, with significant negative correlations in the Flores Sea (*r* =  − 0.37), Celebes Sea (*r* =  − 0.32), Banda Sea (*r* =  − 0.26), and Savu Sea (*r* =  − 0.28), while the South Java Sea exhibits only a weak relationship (*r* = 0.13). In contrast, the Arafura Sea shows no statistically significant correlation for either eddy polarity, suggesting weaker sensitivity to large-scale atmospheric forcing in this region. This spatial pattern may reflect the influence of topographic constraints and regional circulation complexity in sub-basins such as the Banda and Flores Seas, although further process-based dynamical analysis would be required to determine the dominant mechanisms.

Taken together, these results indicate that ITCZ latitude variability is significantly associated with eddy occurrence in several dynamically active sub-basins of the MC, while other regions such as the Arafura Sea show limited sensitivity. This spatial heterogeneity suggests that regional circulation complexity and bathymetric steering may influence how large-scale atmospheric variability projects onto mesoscale eddy activity, consistent with previous studies emphasizing the role of ITF-related instabilities and topographic effects in eddy formation^74^.

## Discussion

### $\beta$-plane correction for eddy detection in Maritime Continent basin

Incorporating the $\beta$-plane formulation significantly improves the understanding and dynamical representation of mesoscale eddies across the MC (Figs. 2 and 3). The variation of the Coriolis parameter with latitude is particularly important in narrow straits and semi-enclosed basins, such as the Makassar Strait, Halmahera Sea, and Banda Sea. Traditional $f$-plane approximations tend to underestimate latitudinal variations in eddy rotation and propagation, whereas $\beta$-plane corrections sharpen azimuthal shear, enhance vorticity signatures (Fig. 7), and reveal deformation zones previously muted^91,92^.

Our results show that meridional migration of eddies and their interaction with bathymetric constrictions strongly modulate eddy intensity and residence time (Yuan et al., 2025). For example, southward-propagating AE in the Makassar Strait experience increased $f$-values (Fig. 8), altering their vorticity budget and propagation speed^69^. Similar structural asymmetries are evident in the Banda and Halmahera Seas, reflecting combined $\beta$-effects and topographic constraints^93^. This framework underscores how planetary vorticity gradients mediate energy exchanges between eddies and the mean flow, thereby influencing ITF pathways and contributing to spatial circulation heterogeneity across the MC.

### Atmospheric controls on mesoscale eddy variability in the Maritime Continent

The ITCZ represents a dominant mode of tropical atmospheric variability, and its meridional migration generates pronounced changes in surface wind patterns and wind stress curl (Fig. 15). Previous studies have demonstrated that ITCZ dynamics can produce sharp, localized enhancements in Ekman pumping associated with strong meridional boundary layer inflow, with peak Ekman pumping occurring near the ITCZ core^94,95^. Such localized wind-driven vorticity forcing provides a physically plausible pathway through which ITCZ variability can modulate upper-ocean conditions relevant to mesoscale eddy formation.

Wind stress curl, through Ekman pumping, is a primary mechanism shaping vertical ocean circulation and influencing the likelihood of CE and AE development^96^. Because the ITCZ structures the meridional gradient of surface winds, its latitudinal migration reorganizes the wind-stress-curl dipole and modulates atmospheric conditions favorable for eddy generation^97^,^63^. Within this physical framework, the hemispheric asymmetry identified in our eddy–ITCZ relationships suggests that atmospheric forcing associated with ITCZ migration may preferentially modulate eddy variability in the NH sector of the MC basin (Fig. 16). This interpretation is consistent with dynamical perspectives in which local wind stress curl forcing plays a dominant role in shaping low-latitude circulation variability, while remote forcing signals provide a secondary modulation of regional oceanic conditions (Lehmann et al., 2002; Kersalé et al., 2021). Given the close spatial alignment between ITCZ-related wind stress curl anomalies and eddy hotspots within the Indonesian Seas, the observed correlations support the view that local wind-driven vorticity forcing represents a likely contributing mechanism, rather than a sole driver, linking ITCZ variability to mesoscale eddy activity (Table 2).

Notably, the sub-basins exhibiting the strongest eddy–ITCZ correlations, such as the Banda and Flores Seas, also coincide with regions where latitude-dependent rotational dynamics are particularly important (Fig. 17). As highlighted earlier, $\beta$-plane corrections enhance the representation of eddy vorticity and azimuthal shear in narrow straits and semi-enclosed basins of the MC. This suggests that resolving the atmospheric modulation of eddy variability through ITCZ-related forcing is closely linked to the dynamical framework used to capture mesoscale eddy structure in low-latitude, *β*-dominated environments.

Previous results indicate that eddy activity in the MC exhibits a clear seasonal modulation associated with the monsoon cycle: CEs occur most frequently during DJF under the northwest monsoon, whereas AEs peak during JJA under the southeast monsoon. Because this monsoon-based analysis was not separated by hemisphere, a more direct comparison with the hemispheric ITCZ relationships can be drawn from the seasonal KDE distributions (Fig. 6 for AEs and CEs).

For AEs, the KDE maps show that during the JJA peak season, eddy density becomes particularly concentrated in the northern sector, especially over the Pacific waters north of Papua. This spatial pattern is consistent with the regression results, which indicate a significantly positive relationship between ITCZ latitude and AE occurrence in the NH (*r* =  + 0.54 annually), with the strongest correlations emerging during MAM. This suggests that the northward migration of the ITCZ is associated with enhanced AE occurrence in the NH, while the climatological maximum in JJA reflects the seasonal background state established by the southeast monsoon.

For CEs, although CE occurrence climatologically peaks during DJF, the regression results show that a northward displacement of the ITCZ tends to suppress CE activity in the NH (*r* =  − 0.59 annually; *r* =  − 0.44 in MAM). Meanwhile, the KDE maps (Fig. 6, right panels) indicate that during JJA, CE density becomes more concentrated in the southern sector, extending from southern Nusa Tenggara toward the Arafura Sea. This suggests a seasonal redistribution of CE activity toward the SH rather than a basin-wide intensification.

Taken together, these patterns suggest that monsoon variability provides the dominant seasonal background controlling the timing of eddy occurrence, whereas ITCZ migration introduces an additional meridional modulation that becomes particularly influential during transitional seasons, shaping the hemispheric redistribution of eddy activity across the MC.

### Novelty, implications, and future directions

This study contributes several advances that improve the understanding of mesoscale eddy dynamics in the MC. First, the application of $\beta$-plane correction provides a more physically consistent representation of eddy structure by incorporating meridional variations in the Coriolis parameter. This correction is particularly important in narrow passages and semi-enclosed basins where slight changes in latitude significantly influence vorticity and geostrophic balance. Second, the integration of the WA-OW criteria produces a more selective detection framework that isolates coherent and long-lived eddies while excluding transient features that commonly arise in complex topographic environments.

A third contribution involves the explicit quantification of atmospheric forcing through the role of the ITCZ. The seasonal migration of the ITCZ introduces alternating patterns of wind stress curl that modulate eddy generation differently in each hemisphere. Positive wind stress curl north of the ITCZ enhances CE activity, while negative curl south of the ITCZ strengthens AE. The hemispheric contrast in correlation between ITCZ latitude and eddy occurrences demonstrates that atmospheric convergence zones play an essential role in regulating mesoscale variability. Moreover, the strongest ITCZ-linked signals emerge in dynamically active sub-basins such as the Banda and Flores Seas, highlighting pronounced spatial heterogeneity across the semi-enclosed MC basin. The influence of the ITCZ is further mediated by regional processes that include stratification, bathymetric constraints, and the pathways of the ITF. These interactions create distinct spatial heterogeneity in eddy activity that varies from basin to basin.

A fourth contribution highlights the interconnected behavior of eddies with the mean flow of the ITF and with monsoonal dynamics. Eddy formation and propagation respond to shear zones, topographic boundaries, and seasonal current reversals that collectively shape the transport of heat, momentum, and tracers throughout the region. The presence of long-lived eddies along primary ITF corridors indicates that mesoscale processes actively modulate the efficiency and variability of inter-basin exchange.

The results of this study provide a mechanistic framework that links planetary vorticity gradients, atmospheric forcing, regional circulation, and basin geometry. This integrated perspective offers new insight into how mesoscale eddies influence regional climate expression in the MC. Future research could apply volume transport diagnostics, surface heat flux analyses, and high-resolution modeling to better quantify the dynamical coupling among eddies, monsoon forcing, and ITCZ variability, as well as their contributions to long-term climate modulation.

## Conclusion

This study presents a refined assessment of mesoscale eddy activity across the semi-enclosed basins of the MC by integrating a $\beta$-plane correction into eddy detection and applying a hybrid WA–OW method. Validation against tide-gauge observations confirms that satellite-derived SLA reliably captures basin-scale variability ($r$ 0.64–0.98; $RMSE$ 0.05–0.13 m), supporting its application in regions where in situ measurements are sparse. Reduced skill at several island-dense sites emphasizes the need to consider fine-scale topography and coastal processes when interpreting altimetry-derived eddy statistics.

The Indonesian seas, characterized by narrow straits, complex bathymetry, and high SSTs, exhibit highly heterogeneous mesoscale eddy activity. Between 1993 and 2022, the WA–OW hybrid method identified 7,656 AEs and 7,415 CEs as persistent features, with radii predominantly 60–80 km and lifetimes mostly shorter than 21–28 days. Spatial patterns highlight key eddy hotspots in the Banda, Maluku, Celebes, and Savu Seas, while shallow and weakly geostrophic regions such as the Java Sea show minimal activity. Seasonal asymmetries are pronounced: AEs peak during the southeast monsoon (JJA), whereas CEs dominate during the northwest monsoon (DJF), reflecting the combined influence of monsoonal wind forcing, background current shear, and $\beta$-plane–related planetary vorticity gradients.

Kinematic analyses indicate that EKE scales with eddy size, whereas vorticity decreases with increasing radius, consistent with vortex stretching and geostrophic adjustment theory. The $\beta$-plane correction is critical for equatorial waters, preventing contamination by westward-propagating planetary signals and allowing more accurate characterization of eddy polarity, deformation, and divergence. Mesoscale eddies actively redistribute mass, momentum, and heat across the Indo-Pacific gateway, modulate vertical exchanges, and likely influence nutrient fluxes that sustain regional productivity.

Eddy variability in the MC is primarily controlled by three interrelated mechanisms: (1) monsoonal forcing and ITCZ migration, which modulate seasonal timing and intensity of eddy genesis; (2) ITF dynamics, which provide background flow and shear for baroclinic and barotropic instabilities; and (3) bathymetric steering, which shapes eddy formation, propagation, and decay. AEs correspond to SLA highs (warm cores) and CEs to SLA lows (cold cores), confirming geostrophic consistency, while $\beta$-plane correction highlights the role of planetary vorticity gradients in structuring and propagating these features.

By providing a rigorously filtered, $\beta$-corrected eddy dataset, this study not only enhances detection accuracy in a climatically critical tropical region but also clarifies the primary drivers of eddy heterogeneity. The resulting database and methodological framework provide a robust foundation for future research on eddy-mediated transport, energy pathways, and their broader impacts on regional climate, ITF variability, and air–sea interactions within the MC.

## Supplementary Information

Supplementary Information.

## Data Availability

The datasets generated and analyzed during the current study, including the eddy census database and associated derived variables, are available from the corresponding author upon reasonable request. The eddy detection and β-plane correction scripts developed for this study are also available for academic and research purposes upon reasonable request. There are no restrictions on their scientific use.
