# Supplementary material for: [image]-plane correction for eddy detection and the drivers of eddy activity heterogeneity in a semi-closed maritime continent basin
Source: Sci Rep. 2026 Mar 31;16:10653. doi: 10.1038/s41598-026-43244-x (PMC13040061; doi:10.1038/s41598-026-43244-x)
Supplement: Supplementary file 1 — Supplementary Information. [file 41598_2026_43244_MOESM1_ESM.pdf]

β-Plane Correction for Eddy Detection and the Drivers of Eddy Activity Heterogeneity in a Semi-Closed Maritime Continent Basin

Gandhi Napitupulu[1,2,3*], Kadek Krisna Yulianti[4,5*], Aditya Rakhmat Kartadikaria[2,3,6*], Ivonne Milichristi Radjawane[2,3,7], M. Apdillah Akbar[4,5], Maya Eria Sinurat[8], Amir Yarkhasy Yuliardi[8], Ejria Saleh[9], Angelo Constancia Macario[10,11], Faruq Khadami[2], Muhammad Ridwan Ramadhan[3], Moses Napitupulu[12]

[1]Coastal Hazards and Energy System Science Laboratory, Graduate School of Innovation and Practice for Smart Society, Hiroshima University, Hiroshima, Japan
[2]Environmental and Applied Oceanography Research Group, Faculty of Earth Sciences and Technology, Bandung Institute of Technology, Bandung, West Java, Indonesia
[3]Study Program of Oceanography, Faculty of Earth Sciences and Technology, Bandung Institute of Technology, Cirebon, West Java, Indonesia
[4]Indonesian Agency for Meteorology, Climatology and Geophysics (BMKG), Jakarta, Indonesia
[5]Master Study Program of Earth Science, Faculty of Earth Sciences and Technology, Bandung Institute of Technology, Bandung, West Java, Indonesia
[6]Center for Coastal and Marine Area Development, Bandung, West Java, Indonesia
[7]Korea-Indonesia Marine Technology Cooperation Research Center, Jakarta, Indonesia
[8]Department of Marine Science, Faculty of Fisheries and Marine Science, Jenderal Soedirman University, Purwokerto. Central Java, Indonesia
[9]Borneo Marine Research Institute, Universiti Malaysia Sabah, Jalan UMS, 88400 Kota Kinabalu, Sabah, Malaysia
[10]Graduate School of Integrated Sciences for Life, Hiroshima University, Higashi-hiroshima City, Japan
[11]College of Fisheries and Allied Sciences, Zamboanga State College of Marine Sciences and Technology, Zamboanga City, Philippines
[12]Study Program of Naval Architecture and Marine Engineering, Faculty of Engineering, University of Indonesia, Depok, Indonesia
gandhinapitupulu88@gmail.com; kadekkrisnay@gmail.com; a.r.kartadikaria@itb.ac.id

Supplementary Figure S1. Validation of satellite-derived sea level anomaly (SLA) against tide gauge observations at selected stations across the Maritime Continent.

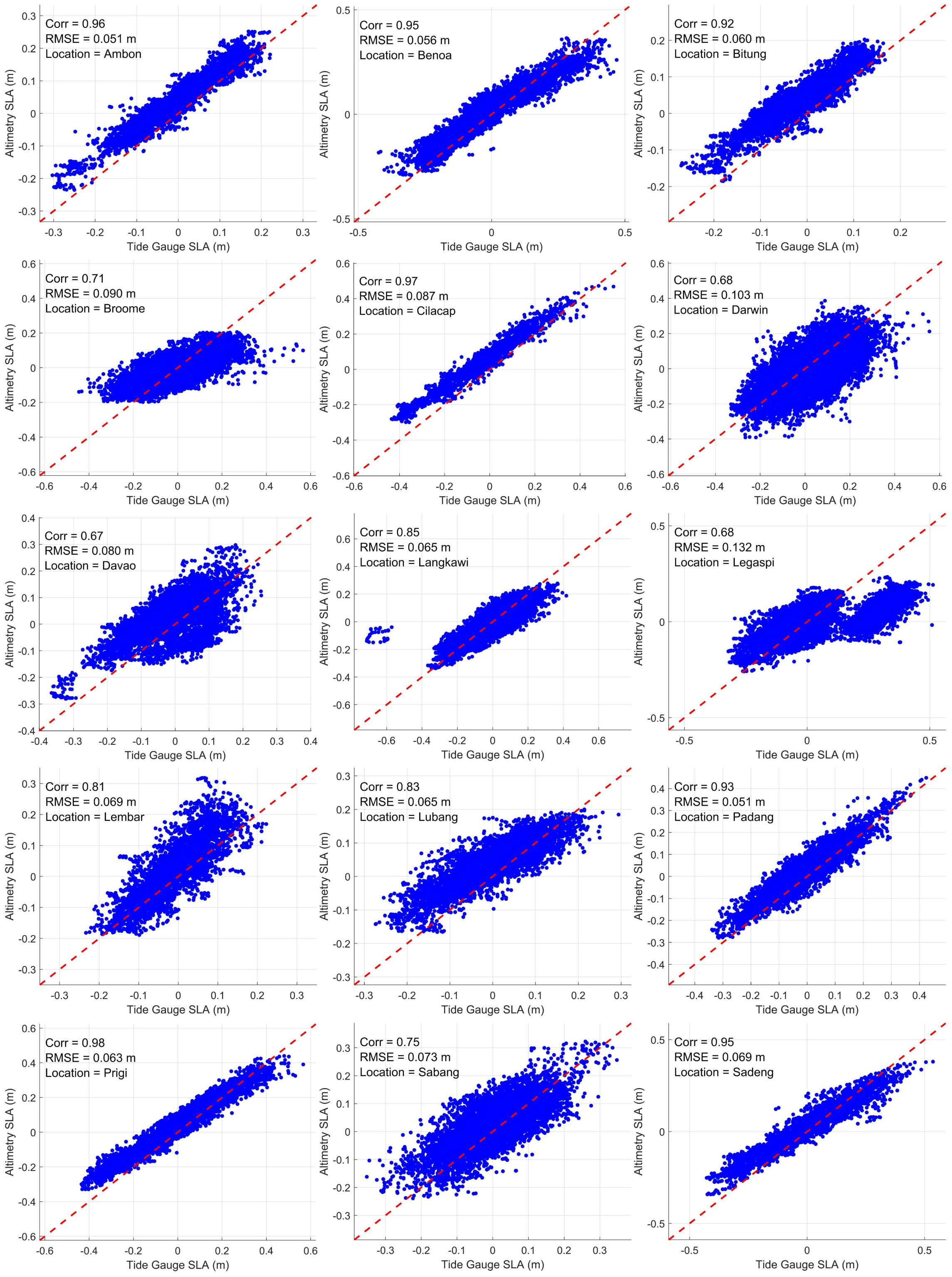

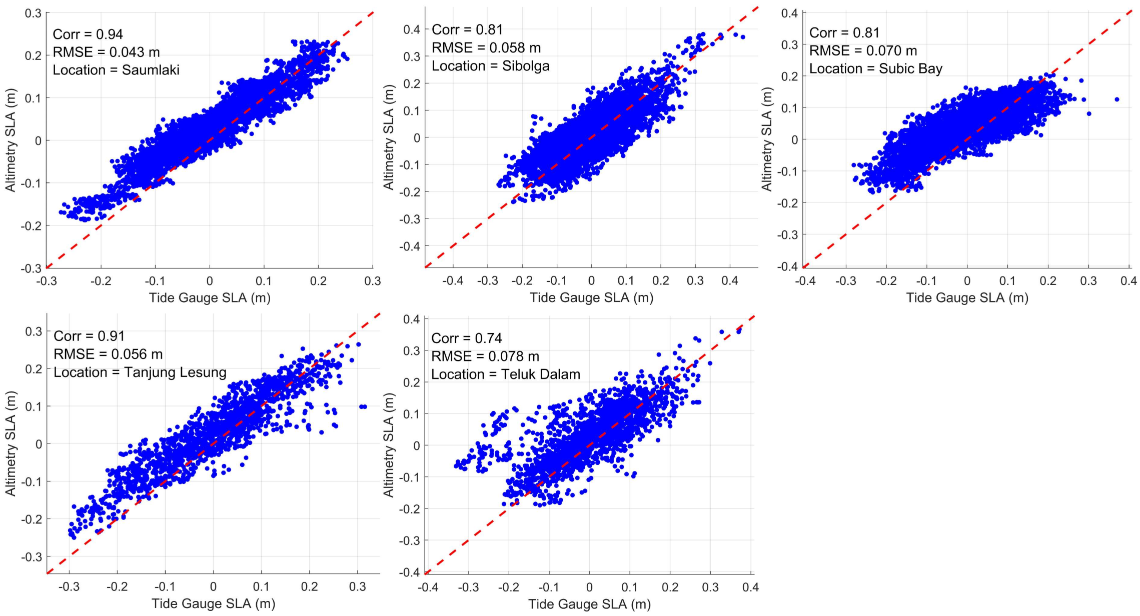
Corr = 0.94
RMSE = 0.043 m
Location = Saumlaki
Corr = 0.81
RMSE = 0.058 m
Location = Sibolga
Corr = 0.81
RMSE = 0.070 m
Location = Subic Bay
Corr = 0.91
RMSE = 0.056 m
Location = Tanjung Lesung
Corr = 0.74
RMSE = 0.078 m
Location = Teluk Dalam
Altimetry SLA (m)
Tide Gauge SLA (m)
